# Rationally designed Ru(ii)-metallacycle chemo-phototheranostic that emits beyond 1000 nm†

**DOI:** 10.1039/d2sc01518h

**Published:** 2022-05-18

**Authors:** Chonglu Li, Yuling Xu, Le Tu, Minhyeok Choi, Yifan Fan, Xiaoqiang Chen, Jonathan L. Sessler, Jong Seung Kim, Yao Sun

**Affiliations:** Key Laboratory of Pesticides and Chemical Biology, Ministry of Education, International Joint Research Center for Intelligent Biosensor Technology and Health, College of Chemistry, Central China Normal University Wuhan 430079 China sunyaogbasp@ccnu.edu.cn; Department of Chemistry, Korea University Seoul 02841 Korea jongskim@korea.ac.kr; Guangdong Provincial Key Laboratory of Luminescence from Molecular Aggregates, South China University of Technology Guangzhou 510640 China; State Key Laboratory of Materials-Oriented Chemical Engineering, College of Chemical Engineering, Nanjing Tech University Nanjing 210009 China; Department of Chemistry, University of Texas at Austin Austin Texas 78712-1224 USA sessler@cm.utexas.edu

## Abstract

Ruthenium complexes are emerging as potential complements to platinum drugs. They also show promise as photo-diagnostic and therapeutic agents. However, most ruthenium species studied to date as potential drugs are characterized by short excitation/emission wavelengths. This limits their applicability for deep-tissue fluorescence imaging and light-based therapeutic treatments. Here, we report a Ru(ii) metallacycle (Ru1100) that emits at ≥1000 nm. This system possesses excellent deep-tissue penetration capability (∼7 mm) and displays good chemo-phototherapeutic performance. *In vitro* studies revealed that Ru1100 benefits from good cellular uptake and produces a strong anticancer response against several cancer cell lines, including a cisplatin-resistant A549 cell line (IC_50_ = 1.6 μM *vs.* 51.4 μM for cisplatin). On the basis of *in vitro* studies, it is concluded that Ru1100 exerts its anticancer action by regulating cell cycle progression and triggering cancer cell apoptosis. *In vivo* studies involving the use of a nanoparticle formulation served to confirm that Ru1100 allows for high-performance NIR-II fluorescence imaging-guided precise chemo-phototherapy in the case of A549 tumour mouse xenografts with no obvious side effects. This work thus provides a paradigm for the development of long-wavelength emissive supramolecular theranostic agents based on ruthenium.

## Abstract 2

Along-wavelength emissive Ru(ii) metallacycle chemo-phototheranostic was prepared *via* self-assembly that exhibits excellent photostability, deep-tissue penetration capability, and promising chemo-phototherapeutic performance *in vitro* and *in vivo*.

## Graphical abstract

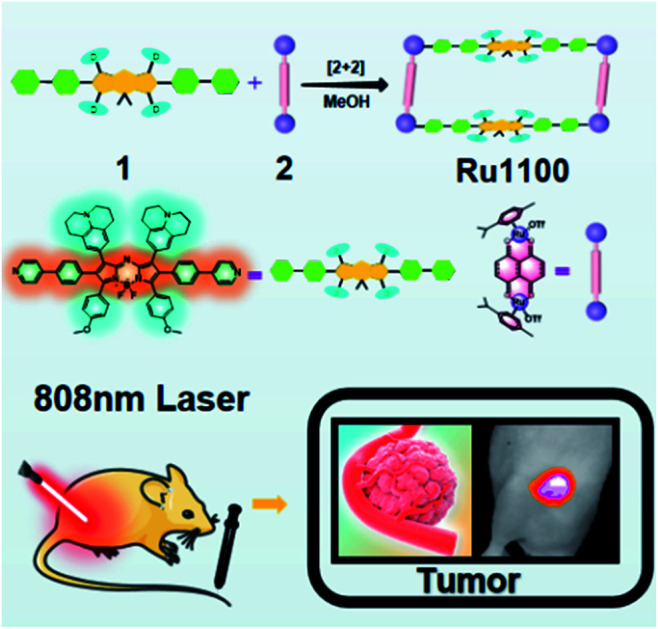

## Introduction

Platinum drugs remain widely used in clinical cancer treatment; however, issues of poor selectivity, dose-limiting side effects, and strong drug resistance continue to animate the search for new therapeutic alternatives.^1–3^ In this context, ruthenium complexes have emerged as promising candidates owing to their high anti-tumour activity, ability to reduce metastasis, and generally low systematic toxicities.^4–7^ For example, polypyridine ruthenium complexes have attracted particular attention as photosensitizers for photodynamic therapy (PDT), of which TLD-1433 has entered into phase II clinical trials for the treatment of bladder cancer.^8,9^ However, most ruthenium complexes studied to date are characterized by short excitation/emission wavelengths (<700 nm), a focus on a single chemo- or phototherapic modality, and unsatisfactory uptake/retention inside cancer cells compared to macromolecular therapeutic agents.^10–13^ These limitations have served to limit the utility of ruthenium-based approaches in the photo-diagnosis or treatment of deep-tissue disease.^14^ These shortcomings, in turn, are providing an incentive to develop new ruthenium complexes that possess longer excitation/emission profiles. We felt this developmental challenge could be met by exploiting a coordination-based self-assembly strategy.

Coordination-driven self-assembly has emerged as useful approach to accessing macromolecules.^15–19^ Among the products of such self-assembly are metallacycles, a number of which have been the subject of drug discovery efforts due to their well-defined shapes, sizes, and their attractive fluorescence and anti-tumour properties.^20–22^ Within this general paradigm, nanoscale-sized Ru(ii) metallacycles have been the focus of particular attention. Such constructs have shown to enter cancer cells selectively and be retained well in such milieus.^23,24^ Furthermore, in many instances the fluorescence performance and phototherapy efficiency of Ru(ii) (and other) metallacycles can be regulated by incorporating appropriate fluorescent ligands.^25–29^ These promising attributes notwithstanding, the use of Ru(ii) metallacycles in applications involving the absorption or emission of light, such as the *in vivo* fluorescence monitoring of therapeutic responses in real-time or imaging-guided therapy, remain relatively unexplored. This paucity of applied utility is largely attributed to the relatively short excitation/emission wavelengths characteristic of most simple ruthenium(ii) complexes (<700 nm as noted above). Recently, we and other groups have worked to develop new agents that permit fluorescence imaging/phototherapy in the so-called second near-infrared biological window (NIR-II, 1000–1700 nm), a spectral region that permits deeper tissue penetration, higher temporal–spatial resolution and better therapeutic outcomes than seen for congeners that absorb or emit in the visible (400–700 nm) or first near-infrared (NIR-I, 700–900 nm) biological windows.^30–36^ We were thus interested to see if these benefits could be extended into the realm of Ru(ii) complexes.

**Scheme 1:**
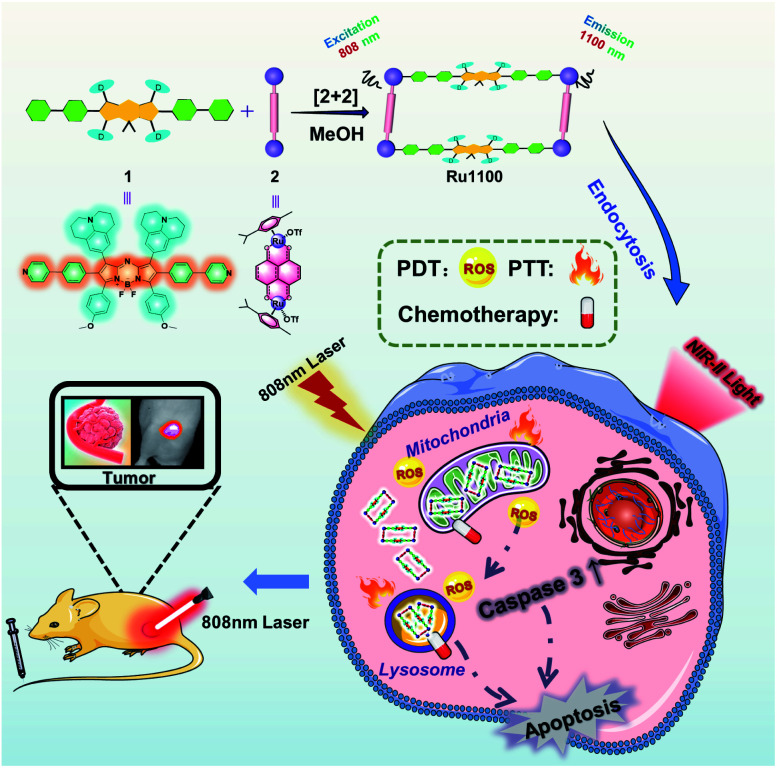
Cartoon illustration of the preparation of Ru1100, mechanisms of Ru1100-induced apoptosis of A549 cancer cells and NIR-II imaging guided tumour diagnosis and therapy.

Given the above considerations, we designed and prepared a long-wavelength emissive Ru(ii) metallacycle (Ru1100, *λ*_em_ = 1100 nm). This metallacycle was obtained *via* the coordination-driven self-assembly of the NIR-II fluorescent ligand 1 (an aza-BODIPY derivative) with the dinuclear arene-ruthenium [Ru_2_(*p*-cymene)_2_(L)OTf_2_] (L = 5,8-dihydroxy-1,4-naphthoquinone) complex 2 (Scheme 1). As detailed below, Ru1100 was found to give rise to NIR-II emission. It also allows for deep tissue penetration (up to 7 mm) and under conditions of red light photo-irradiation displays *in vitro* anti-tumour activity that is superior to that provided by cisplatin and two representative PDT agents, namely 5-aminolevulinic acid (5-ALA) and tris(2,2′-bipyridine)ruthenium(ii) (Ru(bpy)_3_^2+^). After nanoparticle formulation, Ru1100 was found to permit *in vivo* NIR-II fluorescence imaging-guided chemo-phototherapy in the case of an A549 mouse tumour xenograft with no apparent side effects.

## Results and discussion

Based on the calculated highest occupied molecular orbital (HOMO) and lowest unoccupied molecular orbital (LUMO) levels (Fig. S1†), it was considered likely that introducing strong electron donating groups (julolidinyl and anisole) and ruthenium coordination sites (phenylpyridine) into an aza-BODIPY scaffold would allow for the construction of a fluorescent building block, namely ligand 1 (HOMO–LUMO gap = 1.6 eV, *λ*_em_ = 1002 nm). The structure of ligand 1 was characterized by NMR spectroscopy and MALDI-TOF-MS analyses (Fig. S2–S4†). The photophysical properties of ligand 1 were fully characterized by absorption and emission spectra. These studies revealed that the absorption and emission maxima were at ∼820 nm and ∼1000 nm, respectively (Fig. S5†).

**Fig. 1:**
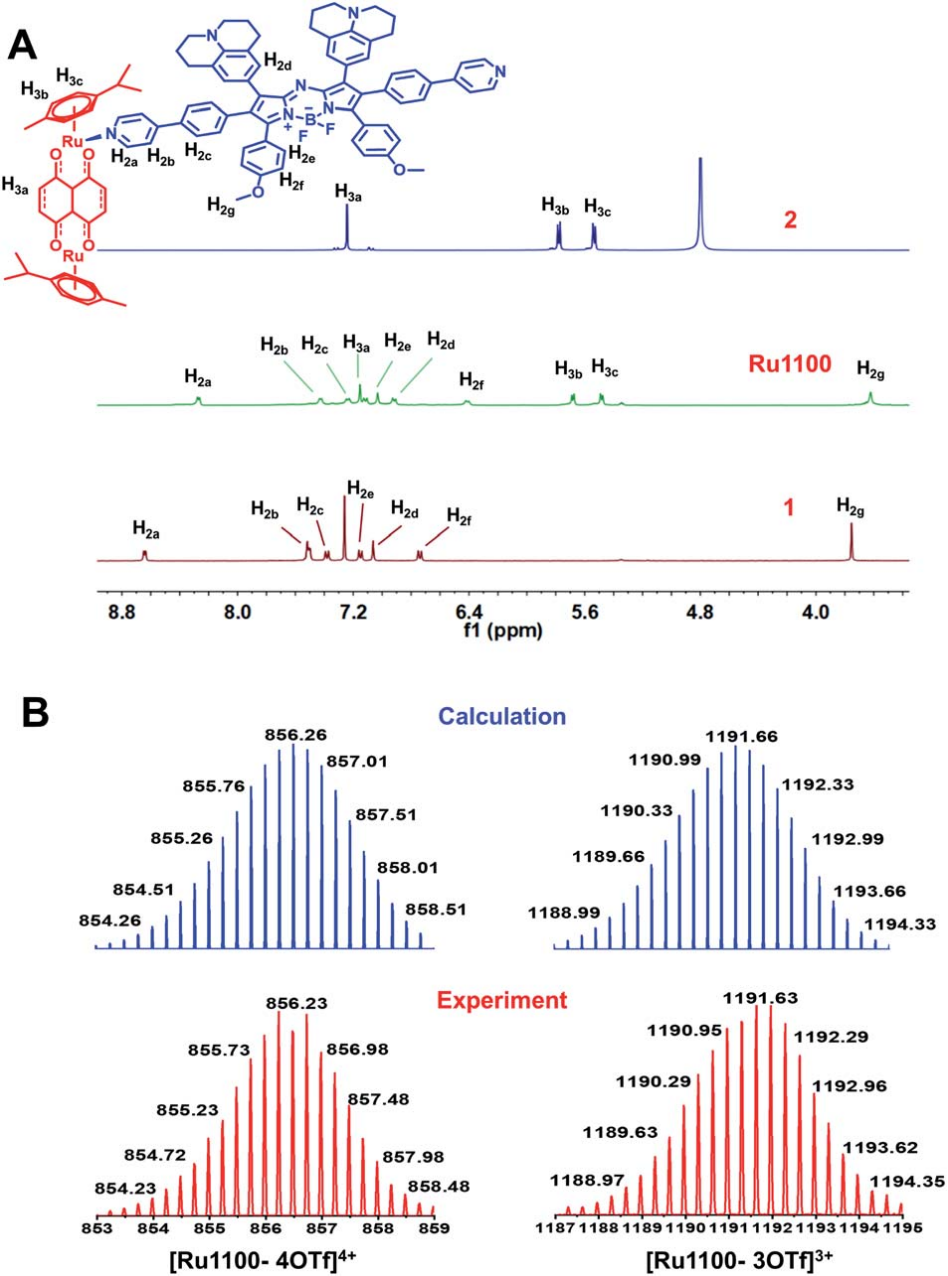
(A) Partial ^1^H NMR (400 MHz, 298 K) spectra of the Ru(ii) complex 2 (top), Ru1100 (middle) and ligand 1 (bottom). (B) Experimental (red) and calculated (blue) ESI-TOF-MS spectra for Ru1100 (left: [Ru1100 – 4OTf]^4+^; right: [Ru1100 – 3OTf]^3+^).

Metallacycle Ru1100 was then prepared *via* the [2 + 2] coordination-driven self-assembly of ligand 1 with the Ru(ii) complex 2 by simply stirring a 1 : 1 mixture in MeOH at room temperature for 24 h (Scheme 1). Characterization was effected by means of ^1^H NMR and ^19^F NMR spectroscopy, electrospray ionization time-of-flight mass spectrometry (ESI-TOF-MS), and 2D-rotating frame Överhauser effect spectroscopy (ROESY). The metal-centred interactions between ligand 1 and the Ru(ii) complex 2 were analysed using ^1^H NMR spectroscopy. As shown in Fig. 1A and S6,† the peak corresponding to the pyridyl proton H_1a_ of Ru1100 was shifted upfield compared to that of the free ligand 1 (*i.e.*, from 8.65 to 8.20 ppm). The protons of the *p*-cymene moiety were also shifter upfield (Δ*δ*[H_3b_] = 0.19 ppm; Δ*δ*[H_3c_] = 0.12 ppm) compared with those of the free Ru(ii) complex 2. The changes in the ^1^H NMR chemical shifts are attributed to the loss of electron density after coordination of the ligand nitrogen atoms to the electron deficient Ru(ii) centre. The ^19^F NMR spectrum of Ru1100 was characterized by a sharp singlet at −79.21 ppm, reflecting the presence of free triflate counter anions (Fig. S7†). Additionally, strong cross peaks between the pyridyl proton of ligand 1 with protons H_3b_ and H_3c_ from the *p*-cymene moiety of Ru1100 were observed in the 2D ^1^H–^1^H ROESY spectrum (Fig. S8†).

ESI-TOF-MS analyses proved consistent with the formation of a rectangular metallacycle (*i.e.*, Ru1100). Specifically, two main peaks readily assigned to the expected [2 + 2] assembly and corresponding to the loss of OTf^−^ counterions (*i.e.*, *m*/*z* = 856.23 for [Ru1100–4OTf]^4+^ and *m*/*z* = 1191.63 for [Ru1100–3OTf]^3+^, respectively) were observed in the ESI-TOF-MS (Fig. 1B and S9†). All the peaks were resolved isotopically and matched well with their calculated theoretical distributions.

Finally, geometry optimizations of the structure of Ru1100 were carried out using the B3LYP functional as implemented in Gaussian 09 (Fig. S10†). On this basis the distance between the two Ru centres in the Ru(ii) complex 2 was calculated to be 8.39 Å; this distance is considerably shorter than the distance between the centroids of ligand 1 (21.19 Å) or that between the two Ru centres coordinated to ligand 1 (24.65 Å).

**Fig. 2:**
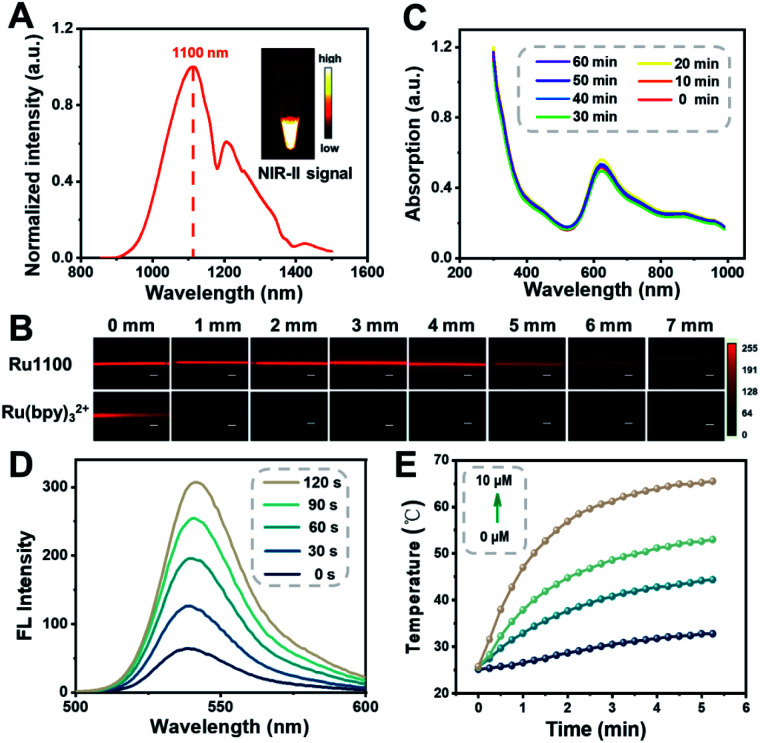
(A) Fluorescent emission spectrum of Ru1100 in CH_2_Cl_2_. Inset: An NIR-II fluorescence image of Ru1100 in CH_2_Cl_2_ (1000 nm long-pass filter) under 808 nm excitation. (B) Fluorescence images of Ru1100 and Ru(bpy)_3_^2+^ encapsulated in capillaries and immersed at varied depths in 1% intralipid. Scale bar: 300 μm. (C) Absorption of Ru1100 in water when subject to 808 nm laser irradiation (0.8 W cm^−2^) for up to 60 min. (D) Fluorescent spectra of Ru1100 (5 μM) + 2′,7′-dichlorodihydrofluorescein diacetate (DCFH-DA; 20 μM) under 808 nm laser irradiation (0.8 W cm^−2^) recorded at different time points. (E) Photothermal curves of different concentrations of Ru1100 when subject to 808 nm laser irradiation (0.8 W cm^−2^) for 0–5 min.

The ultraviolet-visible (UV-Vis) absorption spectra of Ru1100 were recorded in solvents of differing polarities. In all organic solvents tested, the spectra showed a broad absorption band over the 600–900 nm spectral region, which was attributed to intraligand charge transfer (ILCT) within ligand 1 (Fig. S11†).^11^ The emission maximum of Ru1100 was observed at about 1100 nm, which is in the NIR-II region (Fig. 2A). The molar absorption coefficient of Ru1100 in DMSO was 44 890 M^−1^ cm^−1^. This value is greater than that for many reported aza-BODIPY based photosensitizers.^37^ The relative quantum yield (*Φ*_F_) of Ru1100 in DMSO was determined to be 0.062% (using IR26 as a reference) (Table S1†). Moreover, phantom imaging of the NIR-II region revealed that the penetration depth of Ru1100 (in 1% intralipid) could reach 7 mm, *vs.* only 1 mm for Ru(bpy)_3_^2+^ (Fig. 2B).

^1^H NMR and UV absorption spectral analyses served to confirm that the dissociation of Ru1100 in medium was minimal over the course of 24 h (Fig. S12 and S13†). Furthermore, no appreciable change in the absorption spectra of Ru1100 was seen when subject to 808 nm laser (0.8 W cm^−2^) photo-illumination for 60 min, leading us to infer that this complex would not be subject to photo-bleaching when studied either *in vitro* or *in vivo* (Fig. 2C). A complementary UV absorption spectral study revealed that the absorption features of Ru1100 were insensitive to the pH over the 4.5 to 7.5 pH range (Fig. S14†). The classic probe, 2′,7′-dichlorodihydrofluorescein diacetate (DCFH-DA), was used to assess the ability of Ru1100 to produce reactive oxygen species (ROS) upon photo-excitation. As shown in Fig. 2D, DCFH-DA alone was nearly non-emissive, whereas the fluorescence intensity rapidly increased as a function of the photo-irradiation time using an 808 nm laser as the irradiation source in the presence of Ru1100. This finding is taken as evidence that Ru1100 generates ROS effectively.

The photothermal features of Ru1100 were also tested. When subject to photo-excitation (808 nm, 0.6–1.2 W cm^−2^), Ru1100 (0–10 μM) engendered a significant temperature increase and did so in a concentration and power density dependent manner (Fig. 2E and S15†). The photothermal conversion efficiency of Ru1100 was calculated to be 57.9%, a value superior to that reported for the FDA-approved dye, indocyanine green (ICG, 15.8%) (Fig. S16†).^38^ Gratifyingly, Ru1100 was found to display photothermal stability even after seven heating/cooling cycles (Fig. S17†).

**Fig. 3:**
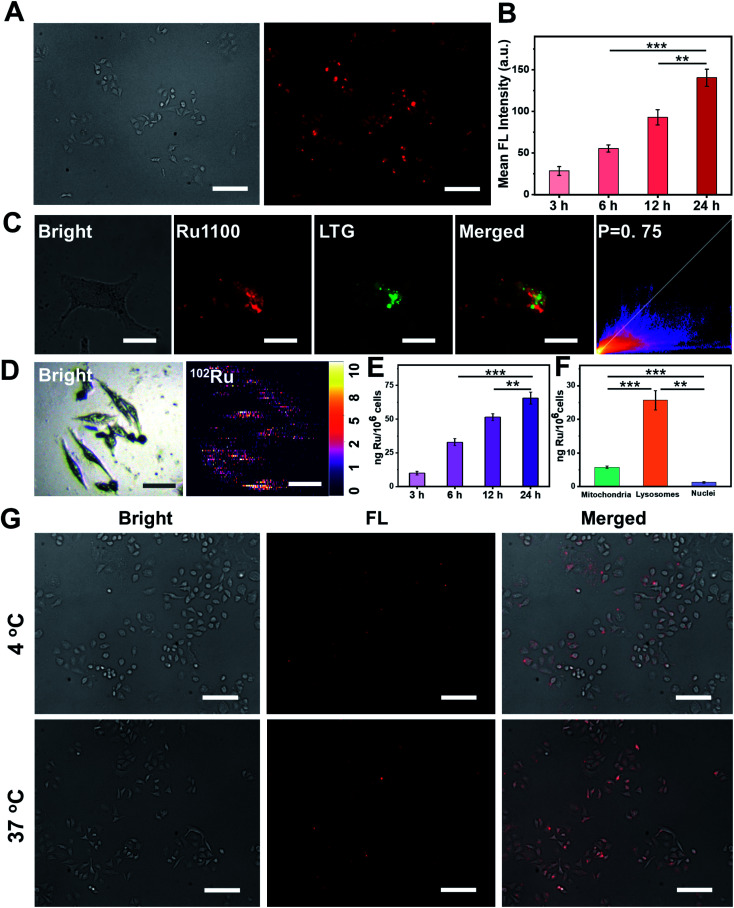
(A) NIR-II fluorescence images of A549 cells incubated with Ru1100 (5 μM) for 24 h at 37 °C. NIR-II images were taken under a 100 ms exposure to a 808 nm excitation source using a NIR-II fluorescence microscope. Scale bar: 300 μm. (B) The average NIR-II fluorescence intensity of A549 cells and the corresponding error bars were obtained by taking the s.d. of their average fluorescence intensity from 20 cells within each NIR-II fluorescence image. (C) Colocalization images of cells treated with Ru1100 and LysoTracker® Green (LTG) and the corresponding correlation coefficients. Scale bar: 10 μm. (D) Elemental distribution in A549 cells as obtained from LA-ICP-MS analyses. Scale bar: 50 μm. (E) Time-dependent ICP-MS of A549 cells pre-incubated with Ru1100 (5 μM). (F) Subcellular distribution and quantification of Ru in A549 cells as inferred from ICP-MS studies. Here, A549 cells were treated with Ru1100 (5 μM) at 37 °C for 6 h in the dark. Lysosomes, mitochondria, and nuclei were extracted using lysosomal, mitochondrial and nuclear isolation kits, respectively. (G) Cellular uptake mechanism studies of Ru1100 (5 μM) at 4 °C and 37 °C, respectively. Scale bar: 300 μm.

Prior research has led to an appreciation that in the case of metallacycles cellular uptake and accumulation is correlated with the lipophilicity/hydrophilicity ratio.^23^ The octanol/water partition coefficient (log *P*_o/w_) of Ru1100 was determined to be 0.741 (Fig. S18†). This complex is thus more hydrophobic than its Ru(ii) complex 2 (log *P*_o/w_ = −0.82). The greater lipophilicity of Ru1100 is attributed to the presence of the hydrophobic ligand 1. In the event, the lipophilicity of Ru1100 was considered conducive to cellular uptake and selective localization. The actual cellular uptake and localization of Ru1100 was tested in a lung cancer cell line (A549). After incubation with Ru1100, the A549 cells displayed a strong intracellular NIR-II fluorescence. As expected, the average fluorescence intensity increased with increasing incubation time (Fig. 3A and B and S19†). Colocalization experiments revealed that the red fluorescence signal arising from Ru1100 overlapped primarily with the green fluorescence from LysoTracker® Green (LTG) after 6 hours incubation (correlation coefficient = 0.75) (Fig. 3C and S20†). A small portion of the Ru1100-based emission was found to overlap with the fluorescence generated from Mito-Tracker® Red (MTR) and Hoechst (Fig. S21†).

The cellular uptake of Ru1100 was further studied by means of laser ablation inductively coupled plasma MS (LA-ICP-MS) and ICP-MS analyses. A strong signal corresponding to ^102^Ru was detected in A549 cells *via* LA-ICP-MS, thus providing additional support for the conclusion that Ru1100 accumulated well in A549 cells (Fig. 3D). The concentration of Ru1100 in cells as determined by monitoring the ruthenium levels *via* ICP-MS was found to increase as the incubation time was extended from 3 h to 24 h, reaching 65.59 ng per million cells at 24 h (Fig. 3E). ICP-MS was also used to determine the subcellular distribution of Ru1100 after a 6 hour incubation period. These analyses revealed that 78.5% and 17.6% of the total Ru was found in the lysosomes and mitochondria, respectively. In contrast, relatively little Ru was found in the nucleus (Fig. 3F).

To gain insight into the putative internalization mechanisms for Ru1100, cellular uptake studies were performed at low temperature in the presence of metabolic and endocytic inhibitors. When A549 cells were pre-cooled to 4 °C, the cellular uptake of Ru1100 was drastically reduced. Such a result is consistent with the entrance of Ru1100 into cells being mediated *via* one or more energy-dependent processes. Incubating the cells with triethylamine, chloroquine, or ammonium chloride had no apparent effect on the internalization of Ru1100, leading us to conclude that the cellular uptake of Ru1100 was not the product of active transport. Pretreatment of cells with sucrose or methyl-β-cyclodextrin resulted in a decrease in the intracellular luminescence intensity. Such a finding is consistent with the internalization of Ru1100 into cancer cells being mediated through the energy-dependent clathrin-mediated and caveolar-mediated pathways (Fig. 3G and S22†).

Based on the above results, the *in vitro* anti-cancer ability of Ru1100 was evaluated by means of a standard 3-(4,5-dimethylthiazol-2)-2,5-diphenyltetrazolium bromide (MTT) assay using several human cell lines, including A549, HeLa, HepG-2, cisplatin-resistant A549 (A549/DDP), and 16HBE cells. Cells were incubated with various concentrations of Ru1100 both in the dark and under laser irradiation (cisplatin and 5-ALA were used as controls) with the results being summarized in Table S2.† Metallacycle Ru1100 gave rise to higher levels of cytotoxicity across all cancer cell lines than the FDA approved drug cisplatin, while its photocytotoxicity index (PI) value was equal to, or greater than, that of the PDT agent 5-ALA. Furthermore, Ru1100 displayed a strong cell killing effect in the case of both the wild type A549 and platinum resistant A549/DDP cell lines. For instance, an IC_50_ value of 1.6 μM was seen in the platinum resistant A549/DDP cell line *vs.* 51.4 μM for cisplatin. A comparison between the A549 cancer cells and the 16HBE normal cells revealed a calculated selectivity index (SI) of 1.7 for Ru1100; this value is considerably better than those of both 5-ALA (0.2) and cisplatin (0.4).

The ability of Ru1100 to retard cancer metastasis was tested using A549 cells through a wound healing assay. A549 cells were grown to confluency, and a wound was created using a pipet tip. As shown in Fig. S23,† cells treated with phosphate buffered saline (PBS) or PBS + laser moved rapidly when monitored over time. This resulted in more than 80% cell growth in the interspace of the wound 48 h post initiation of the test. In sharp contrast, the wound healing percentage was only 3.3% for the Ru1100 + laser group even at 48 h after treatment (Fig. S24†). The potential of Ru1100 to overcome cancer cell invasion was then tested by means of a transwell invasion assay. The results revealed that a large number of cells in the PBS control group invaded to the lower side of the insets, an invasion ratio defined as 100%. Analogous studies involving treatment with Ru1100 or Ru1100 + laser, exhibited low invasion ratios of 38.9% and 26.2%, respectively (Fig. S25†). On the basis of these combined results, we believe that Ru1100 might be able to inhibit effectively the migration of A549 cells, or halt their invasion, thus reducing or eliminating secondary tumour formation *in vivo*.

**Fig. 4:**
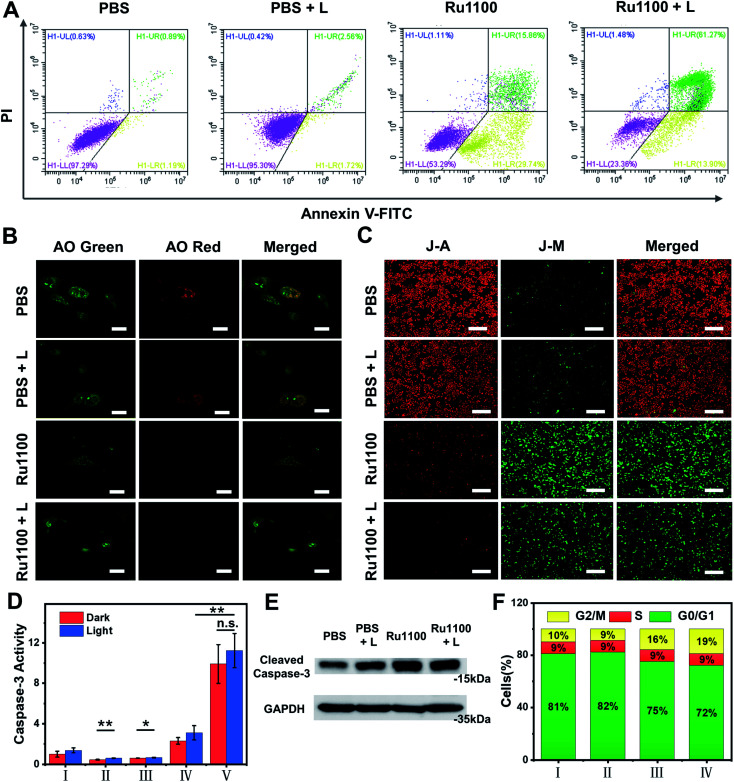
(A) Flow cytometry analysis of annexin-V/PI double-staining of A549 cells. Cells were treated with PBS, PBS + L, Ru1100 and Ru1100 + L, where L represents laser irradiation (808 nm, 0.8 W cm^−2^). (B) Confocal luminescence imaging of acridine orange (AO) stained A549 cells after different treatments. *λ*_ex_: 488 nm; *λ*_em_: 515–545 nm (green channel); 620–640 nm (red channel). Scale bar: 20 mm. (C) Confocal images of A549 cells incubated with Ru1100 and JC-1 in the absence and presence of laser irradiation (808 nm). Scale bar: 400 μm. (D) The activity of caspase-3 in A549 cells pre-incubated with different treatments (I: PBS; II: 5-ALA; III: Ru(bpy)_3_^2+^; IV: cisplatin; and V: Ru1100) were tested before and after laser irradiation (808 nm). (E) Western blot analysis of caspase-3 activation before and after laser irradiation (808 nm, 0.8 W cm^−2^). (F) Distribution of cell population in the cell cycle stages of Ru1100 (I: PBS; II: PBS + L; III: Ru1100; IV: Ru1100 + L).

Given the above promise, an effort was made to elucidate the pathways responsible for cell death associated with Ru1100 treatment. An annexin V-FITC/propidium iodide (PI) double staining assay was conducted to distinguish apoptotic from necrotic cells following administration of Ru1100. As shown in Fig. 4A, treatment with Ru1100 under conditions of 808 nm laser photo-illumination led to a *ca.* 67.3% increase in late apoptotic cells *vs.* 15.9% for the group treated with Ru1100 in the absence of photo-irradiation. We thus conclude that the combination of Ru1100 and laser irradiation (808 nm) produces a strong proapoptotic response and that integration of the chemotherapeutic effect inherent in Ru1100 with a phototherapeutic response under conditions photo-irradiation enhances the overall effect.

As noted above, Ru1100 is taken up primarily into the lysosomes. Therefore, acridine orange (AO) was used as an integrity indicator to evaluate lysosomal dysfunction. This probe produces a red fluorescence in the acidic environment characteristic of lysosomes but emits a green fluorescence when it enters the cytoplasm following lysosomal membrane destruction. As shown in Fig. 4B, when A549 cells were treated with Ru1100 either in the dark or under conditions of photo-illumination, the red fluorescence produced by AO largely disappears, indicating lysosomal damage. The 5,5′,6,6′-tetrachloro-1,1′-3,3′-tetraethyl benzimidazolyl carbocyanine iodide (JC-1) detection kit was then used to study the effect of Ru1100 on the mitochondrial membrane potential (MMP) after chemo-phototherapy. Compared with the control groups consisting of PBS and PBS + laser, cells treated with Ru1100 showed obvious green emission before and after laser irradiation. Such findings are consistent with the MMP being reduced and mitochondrial dysfunction being triggered (Fig. 4C and S26†).

Previous research has served to document that caspases-3/7 are overexpressed during apoptosis. We thus carried out an activity test of caspase-3 *via* a caspase-3 assay kit. As shown in Fig. 4D, the caspase-3 activity in cells treated with Ru1100 under conditions of laser photo-irradiation was ∼3.7-fold higher than that seen in the case of cisplatin. In addition, western blot analyses revealed that Ru1100 led to a substantial increase in caspase-3 expression compared with the control group (Fig. 4E). Cell-cycle progression was also studied by flow cytometry. It was found that Ru1100 served to arrest the cell cycle mainly in the G2/M phase. Treatment with Ru1100 and subjecting to 808 nm laser irradiation (5 min) led to the cells accumulating in the G2/M phase (an increase from 8% in the control group to 36% in the treatment group). Conversely, the number of cells in the S phase hardly increased (Fig. 4F and S27†).

In order to improve the blood circulation time and enhance the tumour targeting of Ru1100, it was formulated in DSPE-mPEG5000 to give Ru1100 NPs. The absorption and emission wavelengths of Ru1100 NPs mirrored those seen for Ru1100 (Fig. S28†). Dynamic light scattering (DLS) and transmission electron microscopy (TEM) were used to investigate the size and morphology of the Ru1100 NPs. A single-peaked distribution with an average hydrodynamic diameter of around ∼255 nm was observed in the DLS experiment (Fig. S29†). The TEM data proved concordant with this value. The results of DLS and TEM indicated Ru1100 NPs are of an optimal size for displaying an enhanced permeability and retention (EPR) effect for *in vivo* applications. Good stability was seen for Ru1100 NPs as inferred from DLS studies carried out in phosphate buffered saline (PBS) over the course of 7 days, as well as absorption spectral studies were carried out in foetal bovine serum (FBS) (Fig. S30 and S31†). In addition, it was found that Ru1100 NPs did not engender appreciable haemolysis or damage to red blood cells (Fig. S32†).

**Fig. 5:**
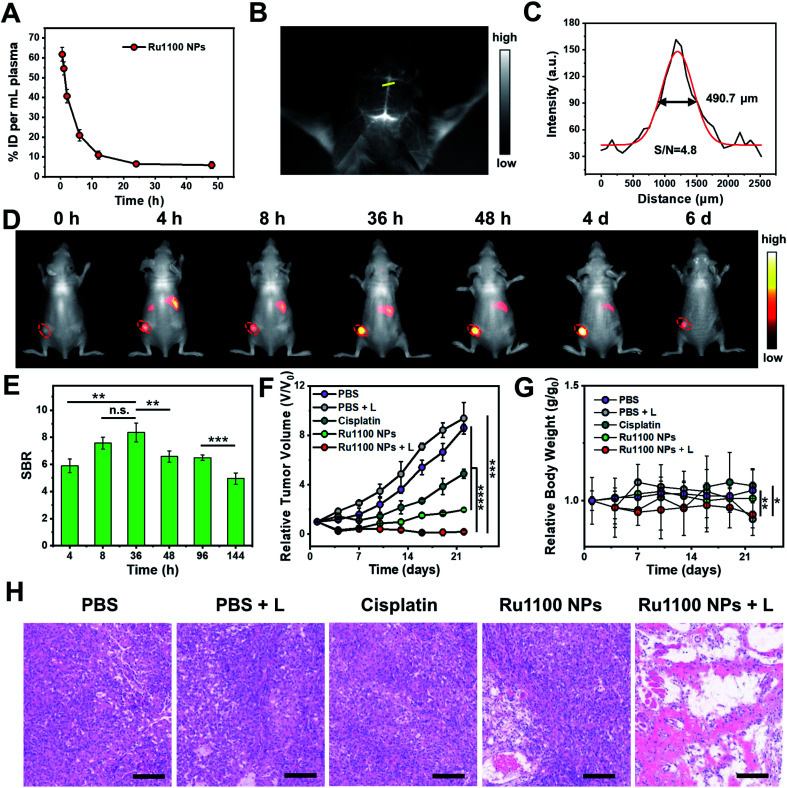
(A) *In vivo* blood elimination kinetics of Ru1100 NP at a dose of 1 mg Ru per kilogram body weight (*n* = 3 for each group). (B) NIR-II images of brain vessels after injection of Ru1100 NPs as visualised under 808 nm laser photo-illumination (28 mW cm^−2^). (C) NIR-II intensity profile (black line) and Gaussian fit (red line) along the yellow dotted line in (B). (D) NIR-II images showing the Ru1100 NP-induced therapeutic response in A549 tumours (28 mW cm^−2^). (E) Normalized fluorescence intensity of (D) (*n* = 4). (F) Tumour growth inhibition after different treatments (*n* = 4). (G) Relative body weight curves after the treatments as in (F) (*n* = 4). (H) H&E staining of A549 tumour slices after the treatments of (F) and (G). Scale bar: 150 μm.

Next, various *in vivo* tests of Ru1100 NPs were carried out. First, the *in vivo* pharmacokinetics of Ru1100 NPs in plasma at different time points were investigated; these studies revealed a relative long blood circulation time as measured by ICP-MS (Fig. 5A). Second, the fact that Ru1100 emits in the NIR-II window led us to explore its potential for cerebral blood vessel imaging. For *in vivo* brain imaging, the skull has a strong scattering effect on light, it is generally necessary to remove the brain skull or grind the skull to make it thinner to reduce the loss of excitation light. These measures may affect the normal function and activity of the brain. It was found that 1 h after injecting Ru1100 NPs (dose: 1 mg Ru per kg body weight) into C57BL/6 mice, visualization of the brain vessel network through the intact scalp and skull could be achieved in a noninvasive manner. A sharp image with a high signal-to-background ratio (SBR = 4.8) was obtained (Fig. 5B and C).

Next, the Ru1100 NPs were tested using a xenograft A549 tumour model. In this case, the tumour could be clearly distinguished at 4 to 48 h post injection by fluorescence with the intensity reaching a maximum 36 h post-injection (SBR = 8.4) (Fig. 5D and E). Additionally, *ex vivo* biodistribution studies, involving both fluorescence imaging and ICP-MS analyses of Ru levels, revealed *in vivo* distribution, tumour accumulation, and hepatobiliary clearance profiles for the Ru1100 NPs that make them attractive for use *in vivo* (Fig. S33 and S34†).

The *in vivo* antitumour activity of Ru1100 NPs as a potential combined chemo-phototherapy agent was assessed using A549 tumour-bearing nude mice. Since the maximum accumulation of Ru1100 NPs occurs around 36 h post-injection as inferred from the above *in vivo* imaging studies, photo-therapeutic studies were initiated 36 h post-injection by exposing the tumour region to 808 nm laser light (0.8 W cm^−2^, 10 min). As shown in Fig. S35,† the temperature of the tumour site in the mice treated with Ru1100 NPs increased to 53.4 ± 0.3 °C within 5 minutes, whereas only a slight increase (to ≤38 °C) was seen in the PBS control group.

After treatment with Ru1100 NP and cisplatin, the tumour volumes of the mice were recorded at 3 day intervals for a total of 22 days. A tumour inhibition level of 78.1% was seen for the Ru1100 NP group. In contrast the corresponding level was 45.6% for the cisplatin group (both *vs.* the PBS control group). Under 808 nm laser irradiation, the tumour inhibition ratio of the Ru1100 NP group was further increased to 97.9% relative to the PBS control subject to identical photo-irradiation (Fig. 5F and S36†).

To evaluate the acute systemic toxicity of Ru1100 NPs, we monitored the body weight changes of the mice. Only modest body weight loss was observed during the above treatments (Fig. 5G). Moreover, no obvious organ damage was observed after treatment with Ru1100 NPs and light as inferred from hematoxylin and eosin (H&E) staining of key organs (Fig. S37†). In addition, H&E staining revealed that chemo-phototherapy caused apparent apoptosis and necrosis in the tumour tissues. These findings are taken as further confirmation of the synergistic anti-cancer effect induced by Ru1100 NPs (Fig. 5H).

## Conclusions

In summary, an emissive Ru(ii) metallacycle that emits beyond 1000 nm (Ru1100) was prepared through coordination-driven self-assembly. Metallacycle Ru1100 showed excellent deep-tissue penetration capability (up to ∼7 mm) and effective chemo-phototherapeutic performance. Once formulated in nanoparticle form, Ru1100 allowed for relatively precise diagnosis and effective therapeutic treatment of a murine cancer model *via* NIR-II fluorescence imaging-guided synergistic chemo-phototherapy under conditions of red laser light (808 nm) photo-excitation. The present study thus serves to introduce a convenient and effective strategy for the development of long-wavelength metal based therapeutic and diagnostic systems of potential medical value.

## Data availability

All data are available in the ESI.†

## Author contributions

J. L. Sessler, J. S. Kim and Y. Sun designed this project; C. Li and Y. Xu synthesized the texted compounds; Y. Xu, L. Tu and Y. Fan conducted *in vitro* studies; C. Li, Y. Xu and M. Choi conducted *in vivo* studies; L. Tu, Y. Fan, X. Chen and Y. Sun analyzed the data; J. L. Sessler, J. S. Kim and Y. Sun wrote the manuscript.

## Conflicts of interest

There are no conflicts to declare.

## Supplementary Material

SC-013-D2SC01518H-s001
