# Supplementary material for: Rationally designed Ru(ii)-metallacycle chemo-phototheranostic that emits beyond 1000 nm
Source: Chem Sci. 2022 May 18;13(22):6541–9. doi: 10.1039/d2sc01518h (PMC9172562; doi:10.1039/d2sc01518h)
Supplement: SC-013-D2SC01518H-s001 [file SC-013-D2SC01518H-s001.pdf]

Electronic Supplementary Material (ESI) for Chemical Science.
This journal is © The Royal Society of Chemistry 2022

Supplementary Information

Rationally Designed Ru(II)-metallacycle Chemo-Phototheranostic that Emits Beyond 1,000 nm

Chonglu Li,[a+] Yuling Xu,[a+] Le Tu,[a+] Minhyeok Choi,[b] Yifan Fan,[c] Xiaoqiang Chen,[d] Jonathan L. Sessler,[e,]* Jong Seung Kim,[b,]* and Yao Sun[a,]*

[a]Key Laboratory of Pesticides and Chemical Biology, Ministry of Education, International Joint Research Center for Intelligent Biosensor Technology and Health, College of Chemistry, Central China Normal University, Wuhan 430079, China.

[b]Department of Chemistry, Korea University, Seoul 02841, Korea.

[c]Guangdong Provincial Key Laboratory of Luminescence from Molecular Aggregates, South China University of Technology, Guangzhou 510640, China.

[d]State Key Laboratory of Materials-Oriented Chemical Engineering, College of Chemical Engineering, Nanjing Tech University, Nanjing 210009, China.

[e]Department of Chemistry, The University of Texas at Austin, Austin, TX 78712, USA.

Table of Contents

S1 Materials/general methods........ S2

S2 Synthesis and characterization of **Ru1100**........ S11

S3 Photophysical properties of **Ru1100**........ S17

S4. Cellular uptake, cell imaging and cytotoxicity studies........ S20

S5. *In vivo* fluorescence imaging guided synergistic chemo-phototherapy........ S26

S6. Synthetic procedures and compound characterization........ S30

S7. Supplementary references........ S44

S1. Materials/general methods

1. General materials and Instrumentation

All chemicals were purchased from commercial sources. ^{1}H, ^{13}C, ^{19}F and 2D ROESY NMR spectra were acquired on a Bruker 400 MHz magnetic resonance spectrometer. Data for ^{1}H NMR spectra are reported as follows: chemical shifts are reported as δ in units of parts per million (ppm) relative to chloroform-*d* (δ 7.26, s); multiplicities are reported as: s (singlet), d (doublet), t (triplet), q (quartet), dd (doublet of doublets), m (multiplet), or br (broadened); coupling constants are reported as a J value in Hertz (Hz); the number of protons (n) for a given resonance is indicated nH, and based on the spectral integration values. Bacteria and cells were purchased from the American Type Culture Collection. The kanamycin-resistant Escherichia coli (kanamycin-resistant *E. coil*) was obtained from Beijing Bio-Med Technology Development Co., Ltd.

MALDI-MS spectrometric analyses were performed on an Applied Biosystems 4700 MALDI TOF mass spectrometer. UV-Vis absorbance was recorded on a PekinElmer Lambda 25 UV-Vis spectrophotometer. The NIR-II *in vivo* system was purchased from Suzhou NIR-Optics Technologies Co., Ltd. NIR-II fluorescence spectra were recorded on an Applied NanoFluorescence spectrometer at room temperature with an excitation laser source of 808 nm. Size distribution and zeta potential were measured via dynamic light scattering (DLS) using a Malvern Nanosizer S instrument. Transmission electron microscopy (TEM) investigations were carried out on a JEM-1200EX instrument.

2. Absorption and photoluminescence excitation (PLE) spectral studies

The UV-Vis-NIR absorbance spectrum of **Ru1100** was recorded on a PerkinElmer Lambda 25 UV-Vis spectrophotometer. PLE spectra of **Ru1100** solutions were obtained using an Applied Nano Fluorescence spectrometer.

3. Measurement of the quantum yield of **Ru1100**

The quantum yield of **Ru1100** was determined according to the formula:

$$\Phi x = \Phi st(\frac{S_X}{S_{st}})(\frac{A_{st}}{A_X})(\frac{n_X^2}{n_{st}^2})$$

to where Φ_{st} is the quantum yield of the standard, S is the area under the emission spectra, A is the absorbance at the excitation wavelength, and n is the refractive index of the solvent used. The x subscript denotes the unknown, and st means standard. 4-(7-(2-Phenyl4H-1-benzothiopyran-4-ylidene)-4-chloro-3,5-trimethylene1,3,5-heptatrienyl)-2-phenyl-1-benzothiopyryliumperchlorate (IR_{26}) was chosen as the standard.[S1]

4. *In vitro* photostability test

The photostability of **Ru1100** was investigated in PBS, with 1 cm quartz cuvettes containing 10 μM **Ru1100** (200 μL) being illuminated using a continuous laser (808 nm, 0.8 W/cm^2) for 60 min. The absorbance of the **Ru1100** solution was recorded every 10 min.

5. Photothermal and photothermal stability tests *in vitro*

Ru1100 solutions of varying concentrations (0, 2.5, 5 and 10 μM) were irradiated with a 808 nm laser (0.8 W/cm^2) for 5 min. In addition, the 5 μM **Ru1100** solution was irradiated with the 808 nm laser at different power densities (0.6, 0.8, 1 and 1.2 W/cm^2) for 5 min. An infrared thermal imaging camera was used to record the solution temperature in 1.5 mL Eppendorf tubes. To test further the photothermal stability of **Ru1100**, solutions of **Ru1100** at a concentration of 5 μM were irradiated with an 808 nm pulsed laser (0.8 W/cm^2) for 5 min and then allowed to cool naturally for 10 min. The solution temperature was recorded over the course of seven heating-cooling cycles using an infrared thermal imaging camera.

6. Measurement of the photothermal conversion efficiency (η)

The photothermal conversion efficiency (η) of **Ru1100** was calculated using the following equations where $T_{\max}$ (or T_{sur}) is the equilibrium temperature (or ambient temperature), I is the incident laser power (I= 0.8 W/cm^2), A_{808} is the absorbance at 808 nm, and τ_s is the system time constant of the sample.

$$\eta = \frac{\text{hs}(T_{max} - T_{sur}) - Q_0}{I(1 - 10^{-A_{808}})}$$

$$\text{hs} = \frac{\sum_i m_i C_{p,i}}{\tau_s}$$

$$\tau_s = \frac{t}{-ln\vartheta} = \frac{t}{-\ln\left(\frac{T-T_{sur}}{T_{max}-T_{sur}}\right)}$$

7. Test of ROS generation *in vitro*

The ROS generation capacity of **Ru1100** was evaluated using a classic ROS indicator DCFH-DA (2',7'-dichlorodihydrofluorescein diacetate).[S2] DCFH-DA (MedChem Express, USA) was dissolved in DMSO (1.0 mM, 0.8 mL) and mixed with NaOH (0.01 M, 2 mL).The resulting deacetylation gave DCFH. The DCFH (20 μM) obtained in this way was added into a DMSO solution of **Ru1100** (5 μM). The solution was then subject to 808 nm laser photoirradiation (0.8 W/cm^2) for 0, 30, 60, 90 or 120 s. The fluorescent spectrum of DCF (λ_{ex}= 488 nm, λ_{em}= 525 nm) was recorded.

8. Octanol/water partition coefficient (log $P_o/_w$)

The partition-coefficient of each compound expressed as

$$logP^{O}_{O/W} = \log\left(\frac{[solute]_{octanol}}{[solute]_{water}}\right)$$

was determined by the "shake-flask" method.[S3] Water and octanol were mixed and shaken thoroughly to reach equilibrium, which resulted in the separation of two layers, i.e., water saturated with octanol and octanol saturated with water. The two layers were separated, and **Ru1100** was dissolved using water that was previously saturated with octanol (3 mL). The same volume of an octanol phase previously saturated with water was then added to the solution. The mixture was shaken at room temperature for 24 hours. The concentration of **Ru1100** and the Ru(II) precursor **2** were determined by UV-VIS spectroscopy using the extinction coefficients of the complexes in water saturated with octanol. The evaluation was repeated three times.

9. Intracellular colocalization monitored by confocal laser scanning microscope (CLSM)

A549 cells were seeded onto 35 mm confocal dishes (Corning) at a density of 1 × 10^4 cells/mL and allowed to adhere overnight. The cells were loaded with **Ru1100** (5 μM, 1% DMSO, *v*%) for 6 h at 37 °C in the dark. The cells were then incubated with LysoTracker®

Green (100 nM), MitoTracker® Deep Red (500 nM) and Hoechst 33342 (5 μg/mL) for 45 min at 37 °C in the dark. The cells were washed with PBS three times at the end of every period. Confocal images were taken using a laser scanning confocal microscope (LSM 710, Carl Zeiss, Germany). For the LysoTracker® Green channel, the excitation wavelength was 488 nm, and the signal was collected between 515-535 nm. For the MitoTracker® Deep Red channel, the probe was excited at 644 nm, and the emission filter was between 665-700 nm. For the Hoechst 33342 channel, the excitation wavelength was 405 nm, and the fluorescence signal was recorded at 430-460 nm. For **Ru1100**, an excitation wavelength of 808 nm was used, and the emission filter was between 1000-1100 nm.

10. Cellular uptake of the ruthenium content measured by ICP-MS

A549 cells were seeded at a density of 1×10^6 cells in 6-well cell culture plates. The cells were left to grow for 24 h in Dulbecco's modified Eagle's medium (DMEM) medium containing 10% FBS and 1% penicillin/streptomycin at 37 °C in 5% CO_2 humidified atmosphere. After 24 h, **Ru1100** (5 μM) was added into the wells and the cells were incubated for 3 h, 6 h, 12 h, and 24 h, respectively. Following incubation, the cells were washed, digested and collected, and the Ru content in the cells determined by ICP-MS. All experiments were repeated three times.

11. Cellular uptake mechanism studies

Cellular imaging was carried out to examine the cellular uptake mechanism.[S4] A549 cells were seeded on 35 mm confocal dishes (Corning) at a density of 1×10^4 cells/mL and allowed to adhere overnight. The culture medium was refreshed with PBS. For the temperature-dependent uptake study, A549 cells were incubated with 5 μM **Ru1100** (1% DMSO, *v*%) for 6 h at 37 °C and 4 °C, respectively. For the cellular uptake inhibition study, triethylamine (1 mM) was used as anion channel inhibitor. Methyl-β-cyclodextrin (50 mM) and sucrose (5 μM) were adopted as caveolin-mediated and clathrin-mediated inhibitors, respectively, while NH_4Cl (50 mM) and chloroquine (100 μM) were used as endocytic inhibitors. A549 cells were pretreated with these protein inhibitors for 40 min at 37 °C, respectively. PBS was then used to wash the cells and the cells were further incubated solely with 5 μM **Ru1100** (1% DMSO, *v*%) for 6 h at

37°C. All of the cells were then washed with PBS three times and subjected to confocal microscopy.

12. Cell viability studies

In vitro cytotoxicity of **Ru1100** was determined by means of MTT assays using several human cell lines, including A549, Hela, HepG-2, A549/DDP and 16HBE. For instance, A549 cells were incubated on a 96-well plate in a DMEM medium containing 10% FBS and 1% penicillin/streptomycin at 37 °C in a 5% CO_2 humidified atmosphere for 24 h with 0.5×10^4 cells seeded per well. Cells were then cultured in the medium supplemented with **Ru1100** at various concentrations for 12 h. The "dark groups" containing **Ru1100** were incubated in the dark for 48 h. For the "light groups", after incubation with **Ru1100** for 12 h, the cells were exposed to 808 nm irradiation for 5 min and then allowed to incubate for an additional 12 h in the dark. The addition of 10 μL of MTT (BioFrox, China) as a 0.5 mg/mL solution to each well was followed by incubation for 4 h at 37°C to allow the formation of formazan crystals. Then, the supernatant was removed and the products were lysed with 200 μL of DMSO. The absorbance value was recorded at 570 nm using a microplate reader. The absorbance of the untreated cells was used as a control and its absorbance was used as the reference value for calculating 100% cellular viability.

13. Wound healing assay

A549 cells were seeded in 12-well plates at a density of 1×10^5 cells/well. The cells were treated with culture medium (negative control, NC), 5-ALA, $[Ru(bpy)_3]^{2+}$, cisplatin, or **Ru1100** (5 μM), respectively. Then, the medium was replaced with PBS, and a scratch was made with a sterile pipet tip (200 μL) in all groups. The detached cells were removed. Laser irradiation was performed in the laser-only group, **Ru1100** + laser group and 5-ALA + laser group. Images were taken immediately after irradiation and 24 h later using a fluorescence microscope. The area of the scratch was analyzed using the ImageJ software.

14. Matrigel invasion assay

Matrigel (Becton Dickinson, Bedford, MA) was added into Transwell inserts (Jet Biofil, China) for solidification in a 24-well plate. Cells were collected and resuspended in serum-free medium and then transferred into the upper chambers, 1×10^6 cells for each Transwell insert. The lower wells were supplemented with 600 μL of the complete medium containing **Ru1100** (5 μM). Photo-irradiation was performed in the PBS + laser group and **Ru1100** + laser group. After 12 h, the cells that did not invade the lower surface of the transwell inserts were cleaned with a cotton swab, while the cells invading the inserts were fixed with 4% paraformaldehyde and subjected to crystal violet staining after being washed with PBS three times. Transwell inserts were visualized by light microscopy. Then, 33% acetic acid was added into the well to dissolve the crystal violet. Cell invasion ratios were calculated according to the absorbance of 33% acetic acid at 590 nm.

15. Annexin V-FITC/propidium iodide double staining assay

A549 cells were seeded in 12-well plates at a density of 1×10^5 cells/well. The dark groups containing **Ru1100** were incubated in the dark for 24 h. For the light groups, after incubation with **Ru1100** for 12 h, the cells were exposed to 808 nm irradiation for 5 min and then allowed to incubate for another 12 h in the dark. Cells in the control group were treated with aculture medium. The cells were further live stained with annexin V-FITC and propidium iodide (PI, Beyotime Biotechnology, China) following the protocols of the manufacturer. Cells were imaged before and after being subject to 5 minutes of laser irradiation (808 nm, 0.8 W/cm^2). Finally, the samples prepared in this way were analyzed via flow cytometry (CytoFLEX, Beckman Coulter).

16. Lysosomes disruption assay

A549 cells were subject to different treatments: 1) untreated; 2) irradiated with 808 nm laser irradiation (0.8 W/cm^2) for 5 min; 3) incubated with 5 μM **Ru1100** for 24 h; 4) incubated with 5 μM **Ru1100** for 24 h and then irradiated with 808 nm laser (0.8 W/cm^2). After treatment, cells were incubated with acridine orange (Macklin, China) at a concentration of 5 μM for 20 min and subjected to confocal luminescence imaging. Confocal luminescence imaging was

performed with excitation at 488 nm and monitoring at 505−545 nm for the green channel or 617-640 nm for the red channel.

17. Analysis of mitochondrial membrane potential (MMP)

MMP was assessed by means of JC-1 staining. A549 cells were seeded onto corning confocal dishes at a density of 1 × 10^4 cells/mL and allowed to adhere overnight. The cells were then treated with culture medium (control) or 5 μM **Ru1100** (1% DMSO, *v*%), respectively. The cells were incubated at 37 °C for 2 h in the dark and then washed with PBS. The cells were then cultured with JC-1 (Solarbio, China) (5 μM) in PBS at room temperature for 20 min in the dark. Fluorescent images were captured by CLSM before and after 808 nm laser irradiation (0.8 W/cm^2). The excitation wavelength for the JC-1 monomer was 488 nm, and the emission filter was adjusted to around 529 nm for the JC-1 monomer (green). For the JC-1 aggregate, and excitation of 543 nm was used, and the emission was collected around 590 nm (red).

18. Caspase-3/7 activation assay

A549 cells were seeded in white-walled nontransparent-bottomed 96-well microculture plates at a density of 1.5 × 10^4 cells/well and allowed to incubate overnight to adhere. The cells were then treated with culture medium (negative control, NC), 5-ALA, $[Ru(bpy)_3]^{2+}$, cisplatin, or **Ru1100**, respectively. The cells were incubated for 12 h in the dark and divided into two equal groups. The dark group was incubated for an additional 12 h and treated with acaspase-3/7 activity kit (Beyotime Biotechnology, China) according to the manufacturer's protocol. The other group was exposed to laser irradiation (808 nm, 0.8 W/cm^2) for 5 min, and incubated for an additional 12 h in the dark. The caspase-3/7 activity was determined using an analogous method.

19. Cell cycle analysis

A549 cells (1 × 10^5) were seeded in 6-well plates and incubated overnight. **Ru1100** (5 μM) were added into different groups. The dark groups containing **Ru1100** were incubated in the dark for 24 h. For the light groups, after incubation with **Ru1100** for 12 h, cells were exposed

to 808 nm irradiation for 5 min and then allowed to incubate for another 12 h in the dark. After treatment, cells were lysed by RNaseA (100 μg/mL, 37°C, 20 min) and stained with PI (100 μg/mL, r.t. 15 min) and analyzed *via* flow cytometry (CytoFLEX, Beckman Coulter).

20. Synthesis of **Ru1100** NPs

Ru1100 NPs were prepared using the matrix-encapsulation method. DSPE-PEG5000 (9 mg in double distilled water) and **Ru1100** (1 mg in THF) were stirred at room temperature overnight. After removing the THF by bubling with nitrogen gas, the resulting mixed solution was centrifuged for 20 min at a speed of 3,000 rpm using a 50 kDa centrifugal filter to remove the residual DSPE-PEG5000 and any free **Ru1100**. Filtering through a 0.22 μm polyester sulfone filter yielded **Ru1100** NPs as an aqueous solution.

21. Hamolysis assay

Fresh blood was obtained in heparinized tubes from mice and subjected to centrifugation at 3000 rpm for 10 min. The blood was washed by PBS (pH = 7.4) three times after adding 20% PBS (v/v) into the blood samples. Stocks of **Ru1100** NPs at different concentrations dissolved in PBS containing 0.2% DMSO (v/v) were made up. Saline was used as a negative control, while water was used as a positive control. Blood was added into Eppendorf (EP) tubes containing **Ru1100** NPs at different concentrations. The mixture was incubated for 1 h by shaking in a water bath at 37 °C. Then the suspension was subject to centrifugation at 3000 rpm for 5 min. After taking photos, the supernatant was added to 96-well plates and the absorbance at 540 nm measured.

Hemolysis Rate (%) = $(OD_{sample}-OD_{saline}) / (OD_{water}-OD_{saline}) \times 100\%$

where OD_{sample}, OD_{saline} and OD_{water} are the absorbances of the sample, positive control and negative control, respectively.

22. *In vitro* NIR-II cell imaging

NIR-II images of the cells were taken at an exposure time of 100 ms using a NIR-II fluorescence microscope. Excitation was effected at 808 nm using a diode laser with an 80 μm

diameter spot focused by a 100 × objective lens (Olympus). The resulting NIR-II fluorescence (FL) was collected using a liquid-nitrogen-cooled, 320 × 256 pixels, two-dimensional InGaAs camera (Princeton Instruments) with sensitivity over the 800 to 1700 nm spectral region. The excitation light was filtered out using a 900 nm long-pass filter and an 1100 nm long-pass filter (both Thorlabs). The NIR FL images were taken at a fixed exposure time of 300 ms. For bright field white-light images, a fiber optic illuminator (Fiber-Lite) was used to illuminate the sample in the trans-illumination mode. Images were recorded using the same filters at a fixed exposure time of 2 ms.

23. *In vivo* NIR-II fluorescence imaging

For brain vessel imaging, a **Ru1100** NPs solution (200 μL, 1mg Ru/kg) was injected into the vein of C57BL/6 mice (n = 4) tail. After injection, the brain vessel system was visualized using the NIR-II imaging apparatus. For tumour imaging, A549 tumour-bearing mice were mounted on the imaging stage beneath the laser. NIR-II fluorescence images were collected using a NIR-II imaging system which was purchased from Suzhou NIR-Optics Technologies CO., Ltd. Photoexcitation was provided by an 808 nm diode laser. The laser power density was 28mW/cm^2 during imaging.

24. *In vivo* antitumour activity

Tumour volume and body weight were measured for animals in all experiments. Tumour volume was determined by measuring the tumour in two dimensions with calipers and calculated using the formula tumour volume = (length × width^2)/2. The mice were divided into four groups randomly (n = 4) when the mean tumour volume reached about 100 mm^3 and this day was set as day 0. Mice were administrated intravenously with PBS, PBS + laser, cisplatin (at a dose of 1 mg Pt per kg body weight), **Ru1100** NPs (at a dose of 1 mg Ru per kg body weight), **Ru1100** NPs + laser. Tumour volumes and body weights were measured every 3 days in the case of the A549 tumour-bearing mice. The tumour inhibition study was stopped on the 22nd day for these A549 tumour-bearing mice.

25. *Ex vivo* biodistribution analysis

Ex vivo fluorescence imaging of organs and tissues was performed with a home-built NIR-II fluorescence imaging system with an InGaAs camera under 808 nm laser diode photo-irradiation (28 mW/cm^2). 24 h after injection of **Ru1100** NPs for NIR-II imaging, the A549 tumour-bearing animals (n = 4) were sacrificed and the liver, kidney, tumour and other organs of each mouse were collected. The NIR-II fluorescent signal of each organ was then measured using the NIR-II imaging system.

S2. Synthesis and characterization of Ru1100

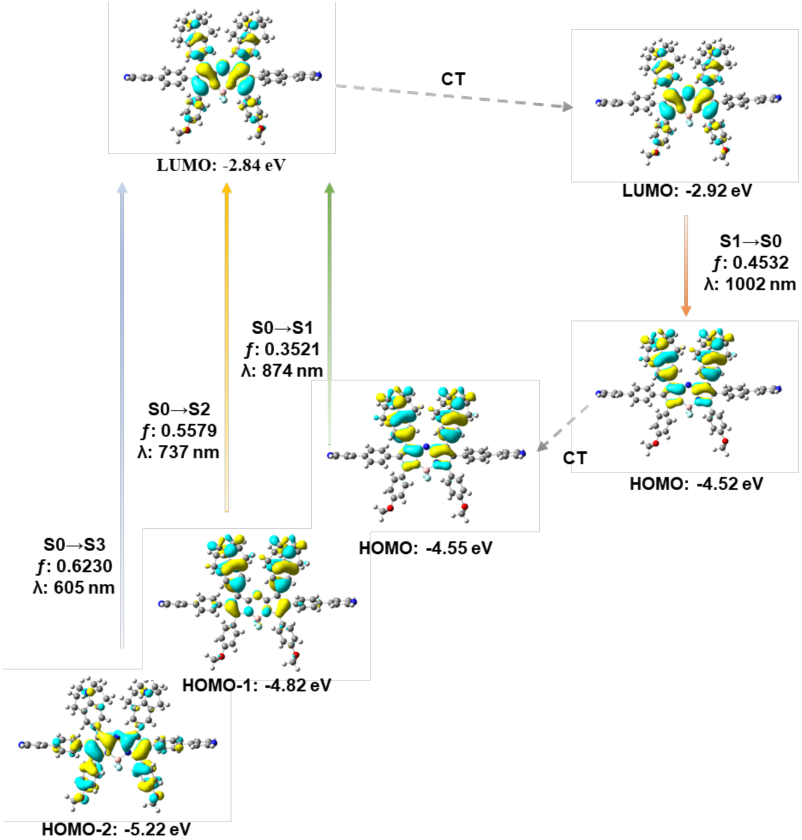

Figure S1. Density functional theory (DFT) calculation of **1**.

Synthesis of ligand **1**:

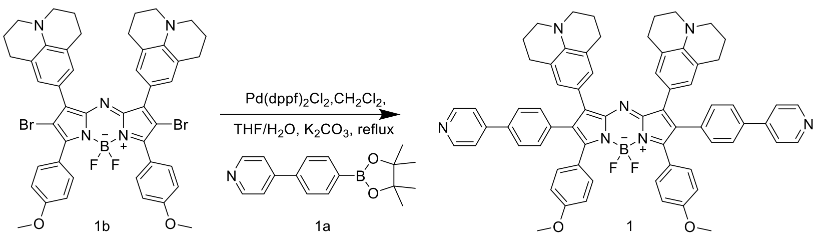

A solution of compound **1b** (20 mg, 0.022 mmol) and compound **1a**[S5] (18.63 mg, 0.066 mmol) in THF (1 mL) was bubbled with argon for 5 min. Potassium carbonate (3.82 mg, 0.027 mmol) in 0.5 mL distilled water and 1,1'-bis(diphenylphosphino) ferrocenepalladium(II) dichloride dichloromethane complex (1.2 mg, 0.0014 mmol) were added to the above reaction mixture under an argon atmosphere. The mixture was heated in an oil bath at 75°C for 8 h. After cooling to room temperature, the volatiles were removed *in vacuo*. The residue was dissolved in dichloromethane, and the resulting solution was washed with water and then saturated aqueous brine. After drying over anhydrous magnesium sulfate and removal of the solvents under reduced pressure, the crude product was purified by silica gel chromatography (pure ethyl acetate, EA, eluent) to afford a blue solid **1** (210 mg, 41.2% yield). ^{1}H NMR (400 MHz, $CDCl_3$) δ 8.64 (d, *J* = 5.3 Hz, 4H), 7.53 – 7.50 (m, 8H), 7.38 (d, *J* = 8.6 Hz, 4H), 7.15 (d, *J* = 7.9 Hz, 4H), 7.06 (s, 4H), 6.74 (d, *J* = 8.5 Hz, 4H), 3.76 (s, 6H), 3.21 – 3.16 (m, 8H), 2.52 (t, *J* = 6.1 Hz, 8H), 1.91 (d, *J* = 5.4 Hz, 8H). ^{13}C NMR (100 MHz, $CDCl_3$) δ 160.18, 149.95, 148.19, 143.49, 140.94, 136.47, 135.63, 132.15, 131.74, 130.55, 126.57, 123.66, 121.36, 120.43, 119.93, 113.16, 55.10, 50.04, 27.55, 21.92.

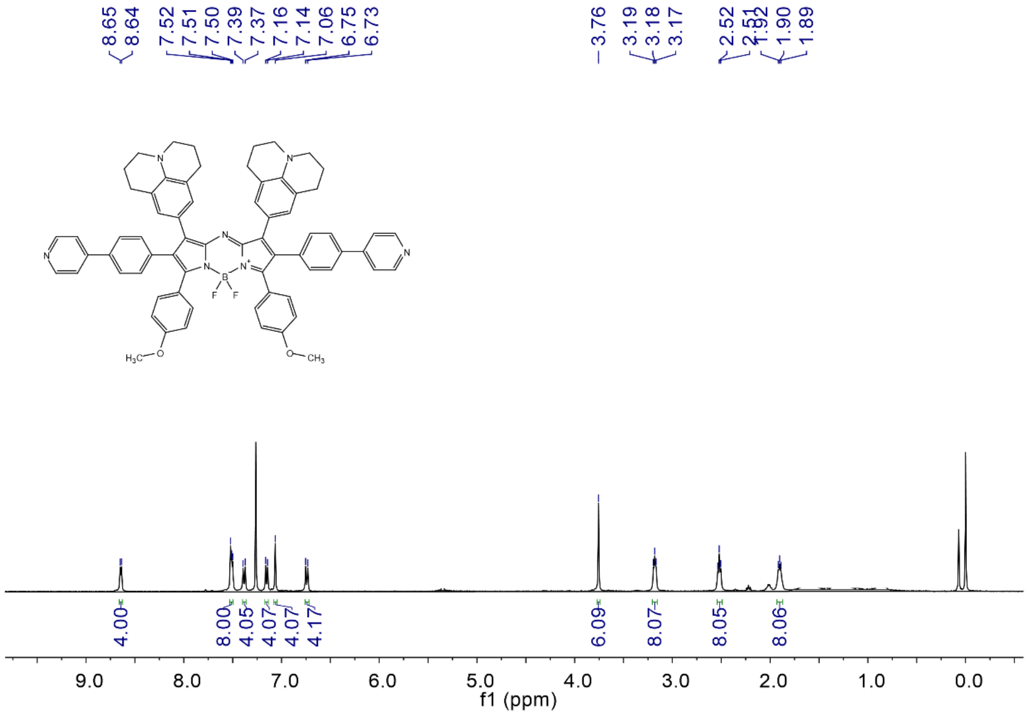

Figure S2. ^{1}H NMR spectrum (400 MHz, $CDCl_3$, 298 K) of **1**.

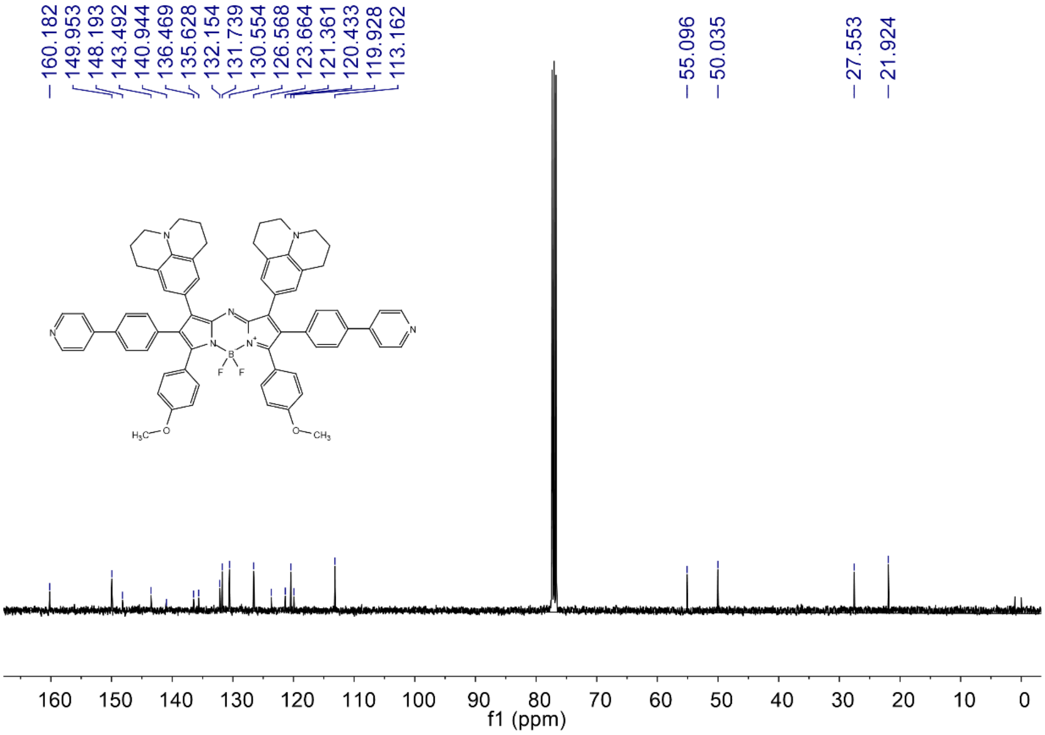

Figure S3. ^{13}C NMR spectrum (100 MHz, $CDCl_3$, 298 K) of **1**.

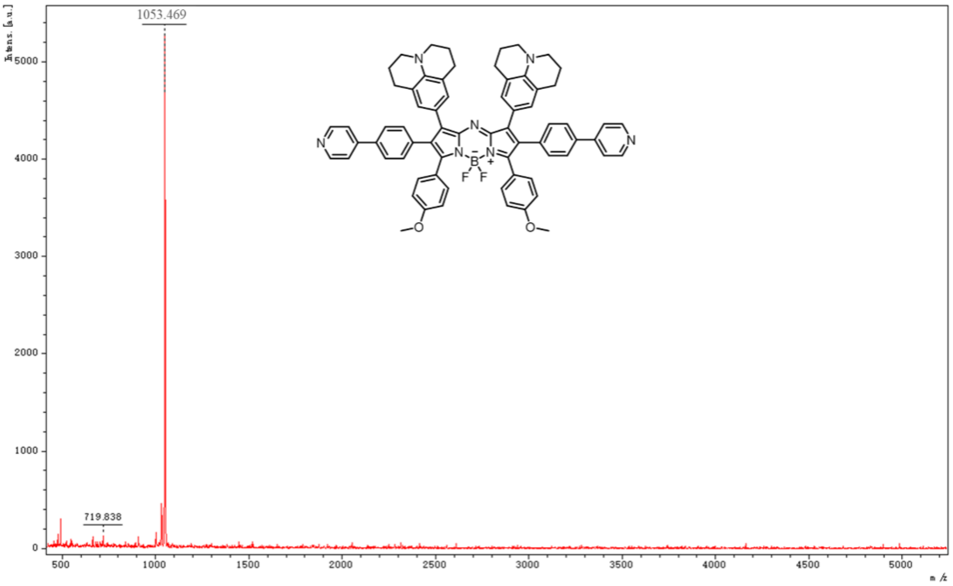

Figure S4. MALDI-TOF-MS of **1**.

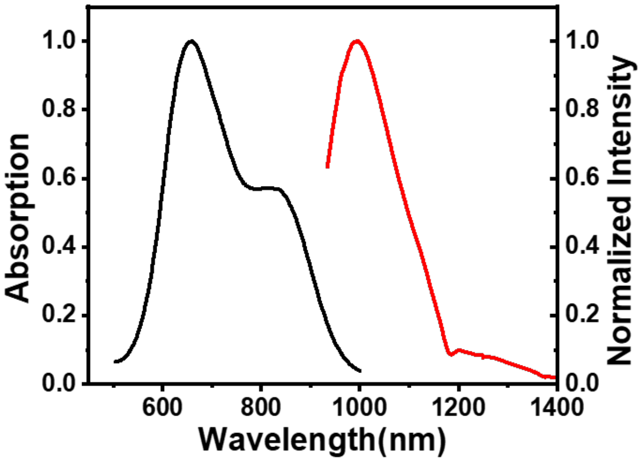

Figure S5. Noremalized absorption and fluorescent emission spectra of **1** in dichloromethane (DCM).

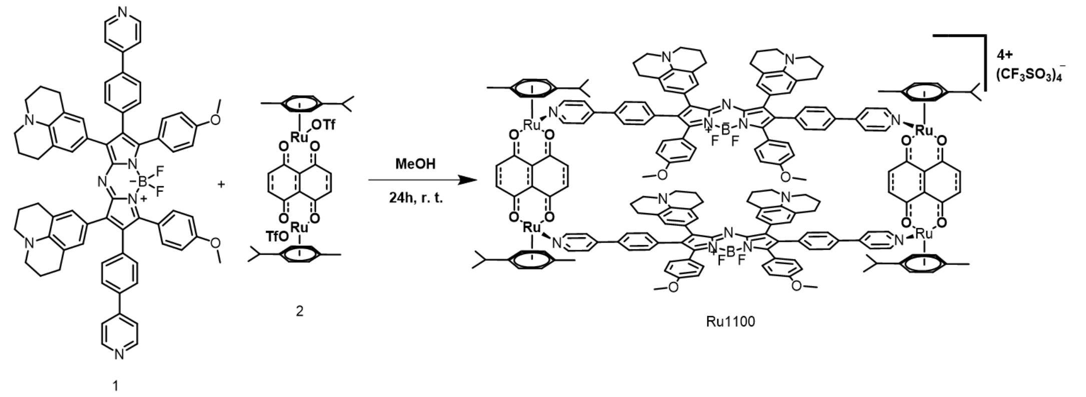

At a 1:1 molar ratio, the ligand **1** (8.0 mg, 0.0076 mmol) and the ruthenium complex **2** (7.26 mg, 0.0076 mmol) were placed in an 8 mL of vial, followed by addition of CH_3OH (6 mL). After stirring at ambient temperature for 24 h, the solution was concentrated to 0.5 mL.The self-assembly products were isolated via precipitation by adding diethyl ether into the concentrated solution, washing twice with diethyl ether and drying under vacuum to obtain product **Ru1100** (11.2 mg, 73.2% yield). ^{1}H NMR (400 MHz, CD_3CN) δ 8.19 (d, *J* = 5.8 Hz, 8H), 7.35 (d, *J* = 5.6 Hz, 8H), 7.16 (d, *J* = 7.7 Hz, 9H), 7.08 (s, 8H), 7.04 (d, *J* = 8.5 Hz, 8H), 6.96 (s, 8H), 6.84 (d, *J* = 7.7 Hz, 7H), 6.34 (d, *J* = 7.8 Hz, 8H), 5.61 (d, *J* = 6.0 Hz, 8H), 5.41 (d, *J* = 6.0 Hz, 8H), 3.60 – 3.51 (m, 12H), 3.13 – 3.05 (m, 16H), 2.73 (d, *J* = 7.0 Hz, 4H), 2.30 (s, 16H), 2.02 (s, 12H), 1.75 (d, *J* = 9.9 Hz, 16H), 1.18 (s, 24H).

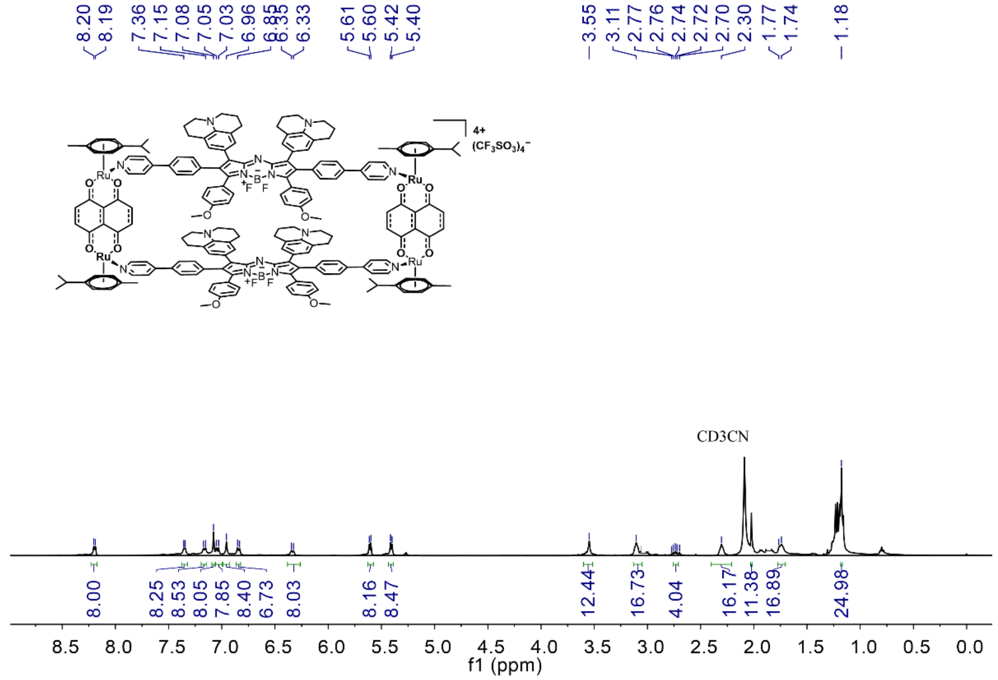

Figure S6. ^{1}H NMR spectrum (400 MHz, CD_3CN, 298 K) of **Ru1100**.

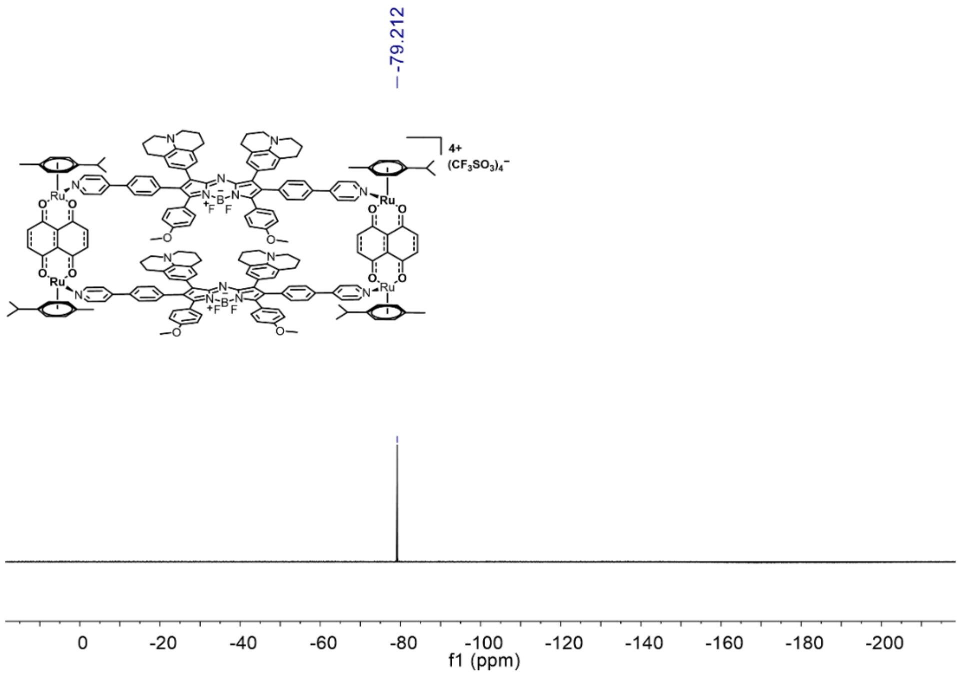

Figure S7. ^{19}F NMR spectrum (400 MHz, CD_3CN, 298 K) of **Ru1100**.

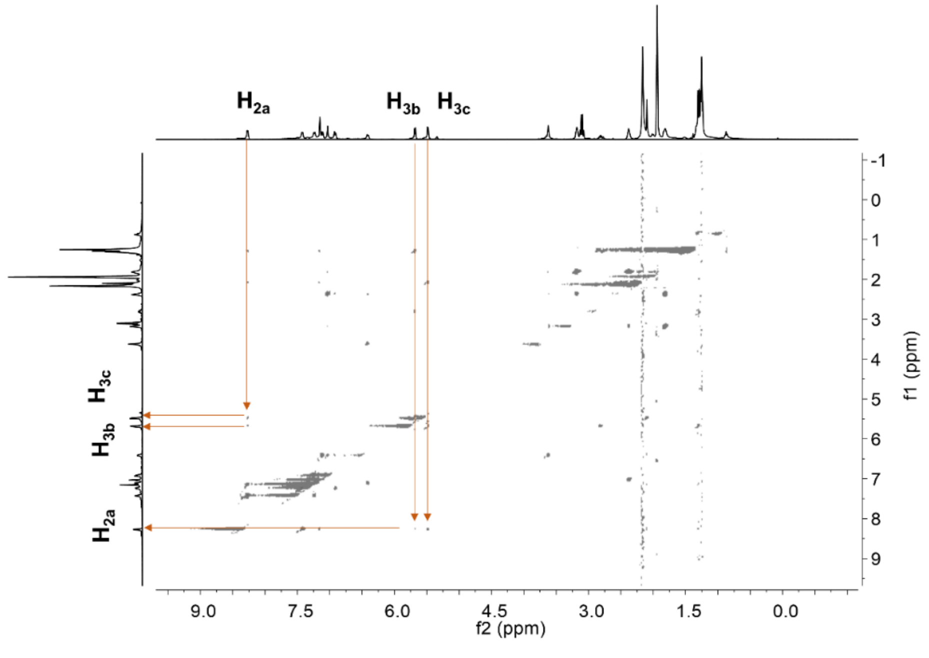

Figure S8. 2D ROESY NMR spectrum (400 MHz, CD_3CN, 298 K) of **Ru1100**.

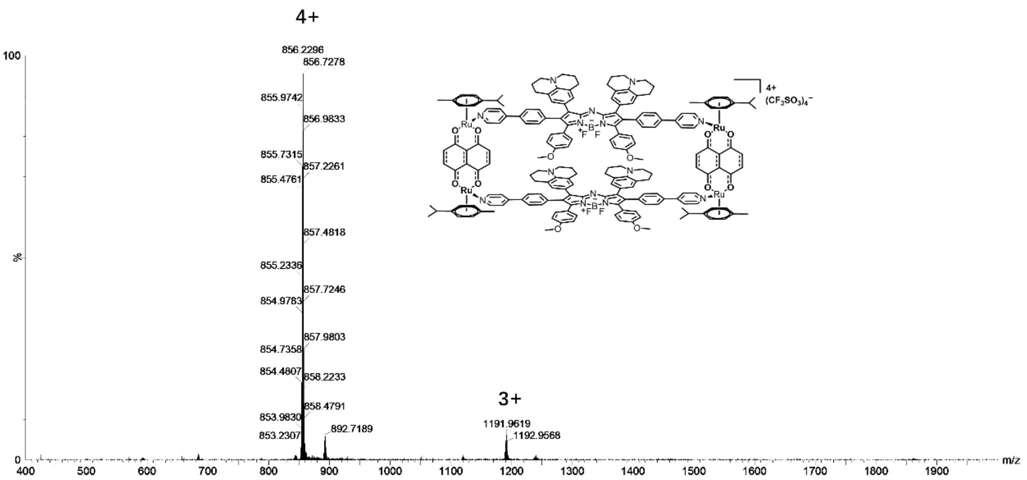

Figure S9. ESI-TOF-MS spectrum of **Ru1100**.

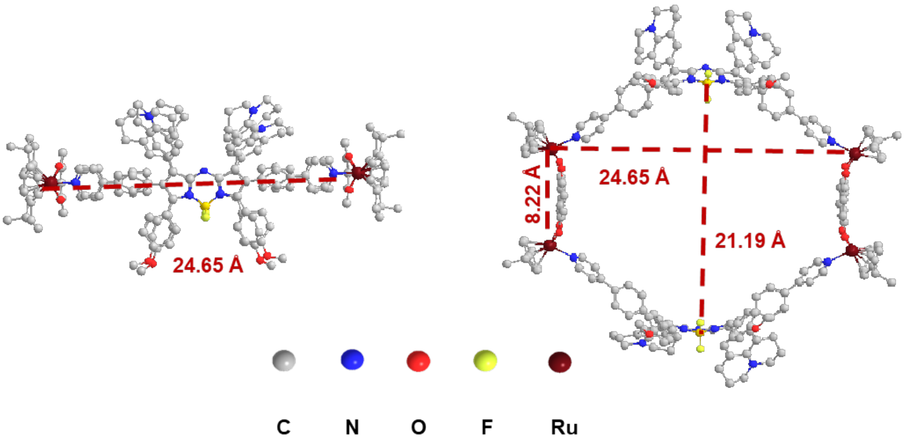

Figure S10. Top and side views of the optimized structure of **Ru1100**. Note: Hydrogen atoms are omitted for clarity

S3. Photophysical properties of Ru1100

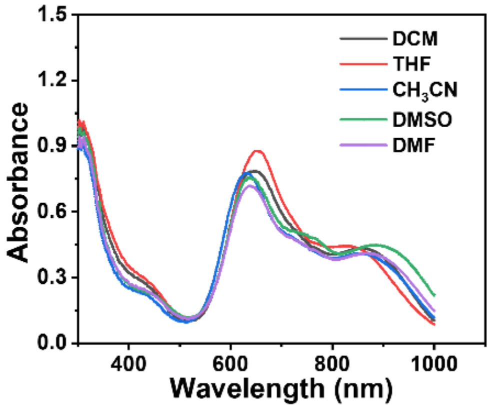

Figure S11. Absorption spectra of **Ru1100** (5 μM) in different solvents.

Table S1. Absorption and emission data for **Ru1100** in different solvents

Solvent	λ_{abs}(nm) [a]	ε_{max}($\times 10^4$ $M^{-1}cm^{-1}$)	λ_{em}(nm) [b]	Φ_f(%) [c]
Dichloromethane(DCM)	857	4.3230	1055	0.072
Tetrahydrofuran(THF)	829	4.4330	1015	0.082
Methyl Cyanide	854	4.0780	1110	0.032
Dimethyl sulfoxide(DMSO)	885	4.4890	1115	0.062
N,N-Dimethylformamide(DMF)	866	4.1200	1100	0.038

[a]Maximum absorbance of **Ru1100**. [b]The maximal emission of **Ru1100**. [c]The relative fluorescence quantum yield relative to IR-26 (0.1% in dichloromethane) used as a reference standard.

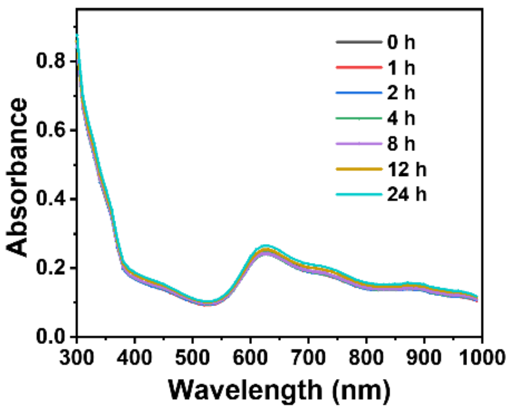

Figure S12. Absorption spectra of **Ru1100** in water recorded at various times.

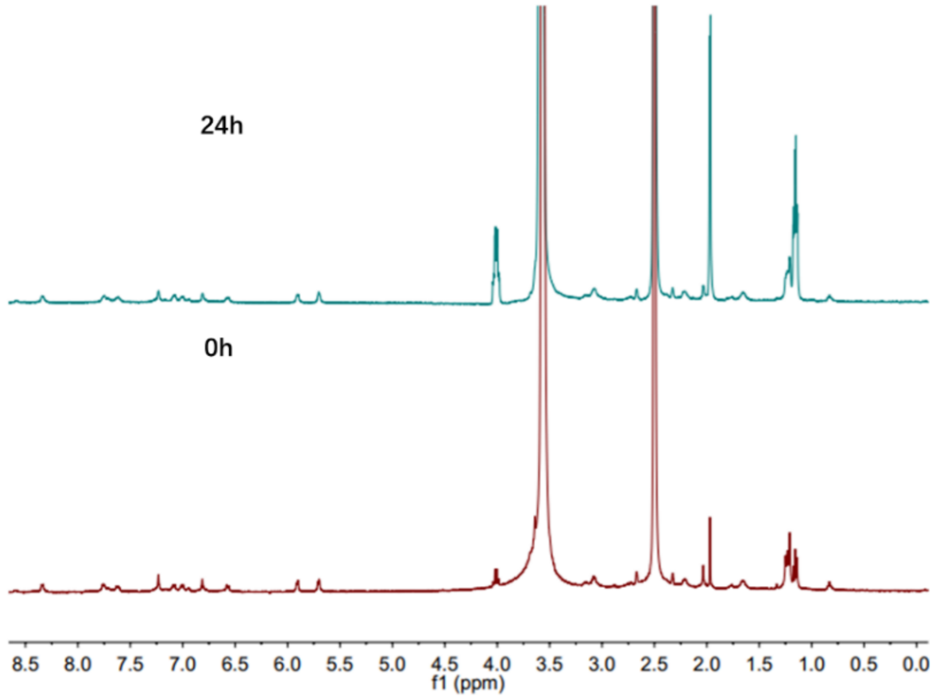

Figure S13. ^{1}H NMR spectra of **Ru1100** at 0 h and after allowing to sit for 24 h in DMSO-d_6 and D_2O (v/v = 4/1).

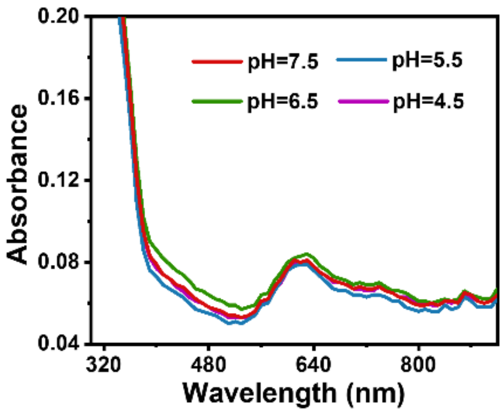

Figure S14. UV−vis−NIR absorption spectra of **Ru1100** (5 μM) recorded at different pH values.

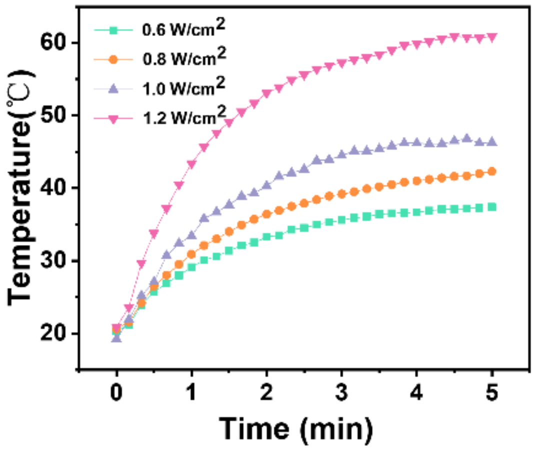

Figure S15. Photothermal curves of **Ru1100** determined at different power densities upon subjecting to 808 nm laser irradiation for 5 min.

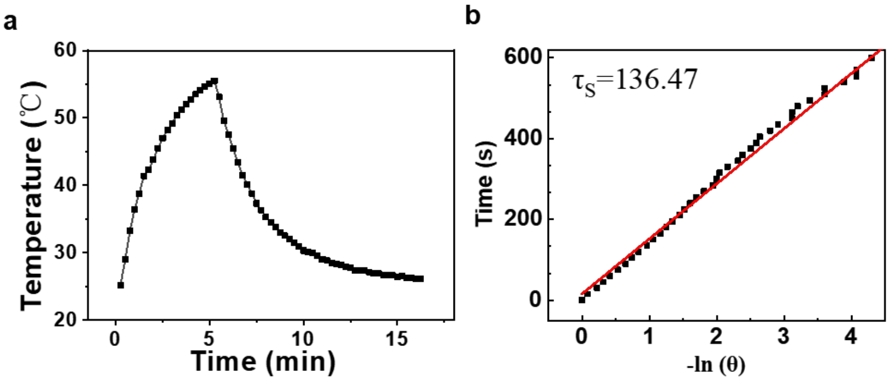

Figure S16. (a) Heating and natural cooling curve of **Ru1100** (5 μM) in DMF under 808 nm laser irradiation (0.8 W/cm^2); (b) Linear time versus -lnθ data from the cooling period of Figure S15a.

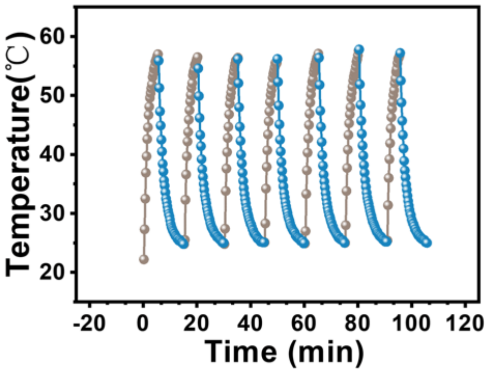

Figure S17. Photothermal heating and natural cooling cycles for **Ru1100** in DMF solution under 808 nm laser irradiation (5 μM, 0.8 W/cm^2).

S4. Cellular uptake and cell imaging studies

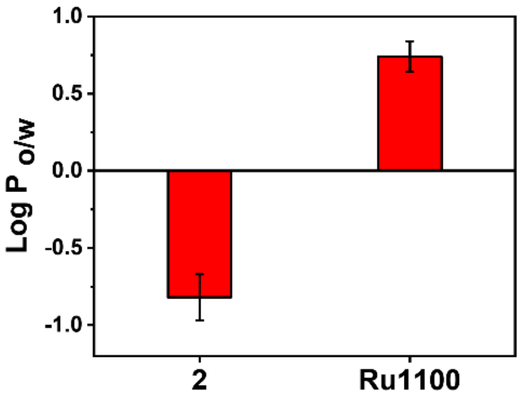

Figure S18. Octanol/water partition coefficients of **Ru1100** and Ru(II) complex **2**. The error bars denote the standard deviation calculated from three replicate trials.

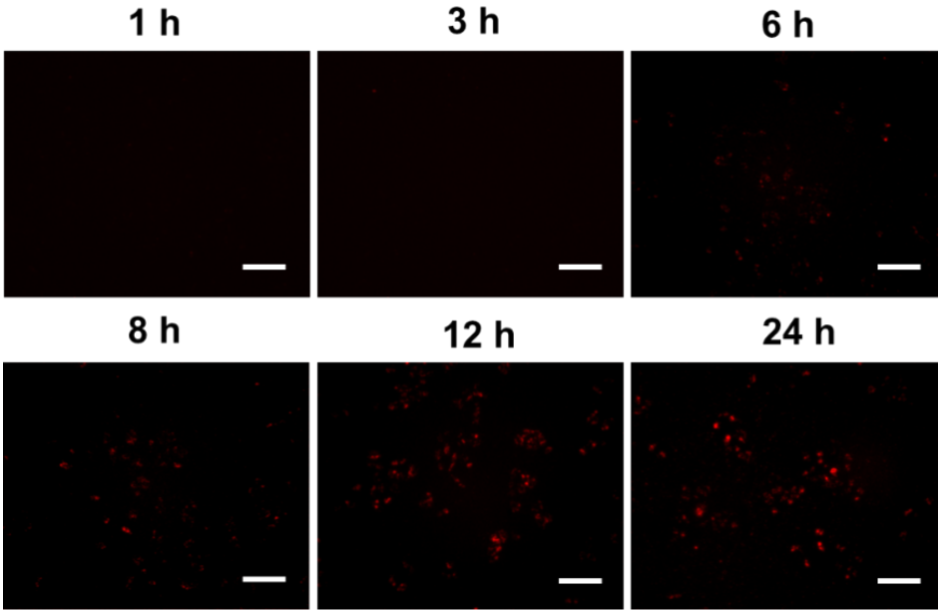

Figure S19. NIR-II fluorescence images of A549 cells incubated with **Ru1100** (5 μM) for different times. Scale bars = 300 μm.

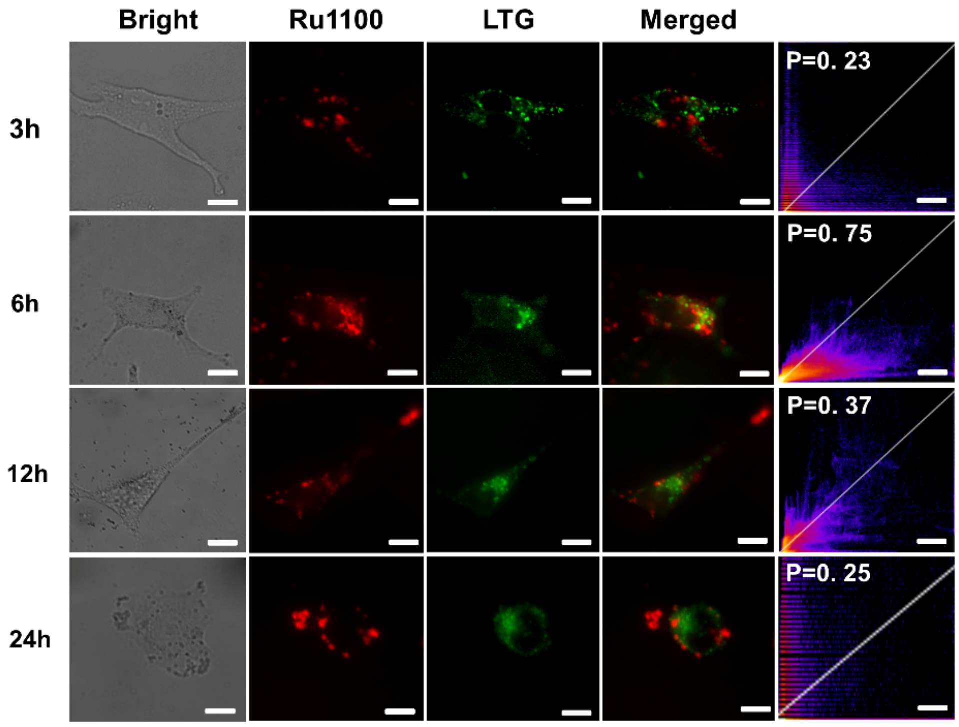

Figure S20. Colocalization images of **Ru1100** (5 μM) and LysoTracker® Green (LTG) at different time points with the corresponding correlation coefficients. Scale bars = 10 μm.

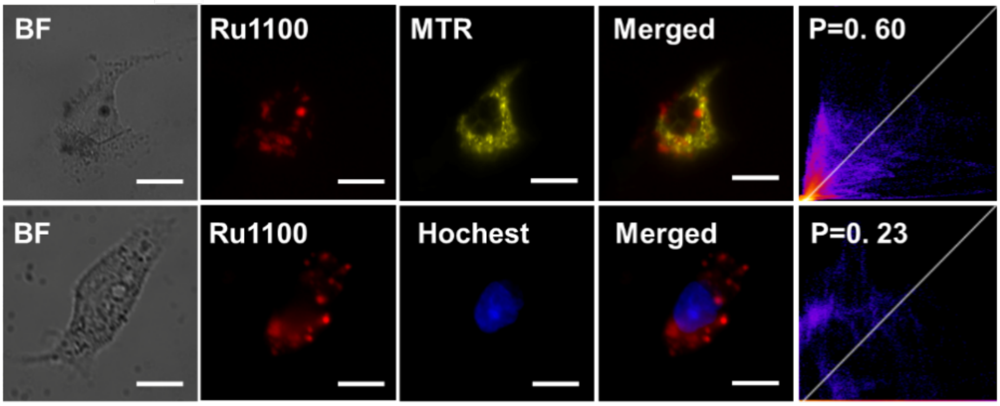

Figure S21. Colocalization images of **Ru1100** (5 μM) with Mito-Tracker® Red (MTR) and Hoechst with the corresponding correlation coefficients. Scale bars = 10 μm.

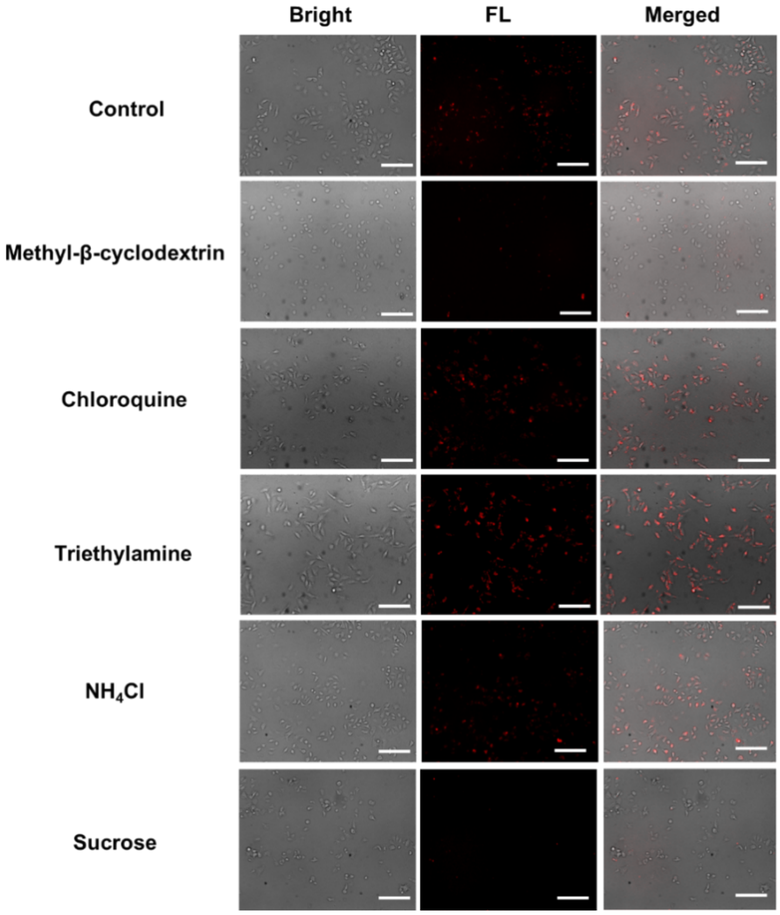

Figure S22. Cell uptake mechanistic study of **Ru1100** (5 μM) in the presence of different inhibitors/conditions. Scale bars = 20 μm.

Cell	Dark/Light	Cisplatin	5-ALA	Ru1100
A549	Dark	21.5 ± 0.1	>300	13.6 ± 5.9
	Light	17.6 ± 4.0	204.7 ± 37.8	4.6 ± 2.6
	PI[a]	1.1	>1.5	2.9
Hela	Dark	24.1 ± 2.7	>300	12.6 ± 3.4
	Light	24.3 ± 2.2	150.9 ± 26.2	6.8 ± 1.4
	PI[a]	<1	>2	1.8
HepG-2	Dark	15.5 ± 9.9	>300	20.4 ± 10.8
	Light	12.7 ± 6.7	115.0 ± 25.4	8.7 ± 2.0
	PI[a]	1.2	>2.6	2.3
A549/DDP	Dark	49.8 ± 3.0	>300	5.4 ± 0.7
	Light	51.4 ± 2.8	135.0 ± 11.8	1.6 ± 2.6
	PI[a]	<1	>2.2	3.4
16HBE	Dark	3.5 ± 1.4	>300	16.8 ± 1.1
	Light	3.3 ± 0.9	72.1 ± 15.6	7.8 ± 1.2
	SI[b]	0.19	0.35	1.7

Table. S2 Cytotoxicity (IC_{50}, μM) of cisplatin, 5-ALA and **Ru1100** toward various cell lines. [a]PI (photocytotoxicity index) = IC_{50} (dark) /IC_{50} (light); [b]SI (selectivity index) is defined as IC_{50} (light) in 16HBE/IC_{50} (light) in A549.

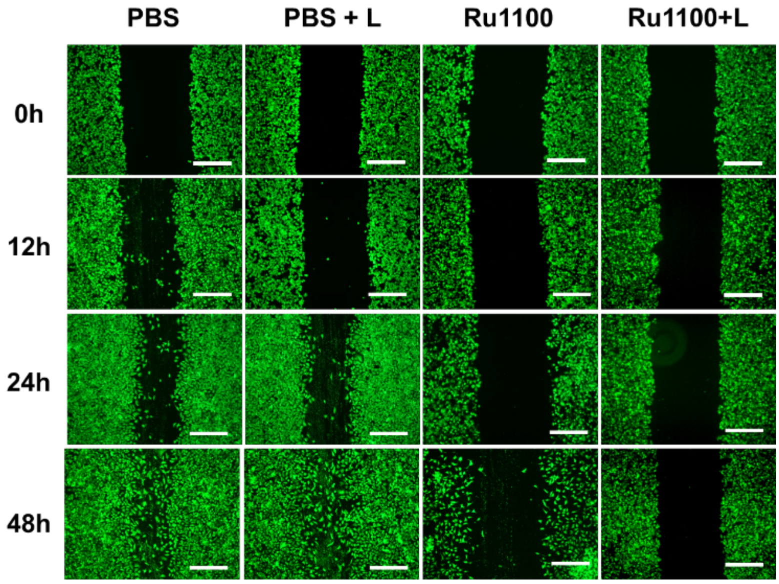

Figure S23. Migration analysis of A549 cells at different time points after subjecting to different treatments. Scale bars = 200 μm.

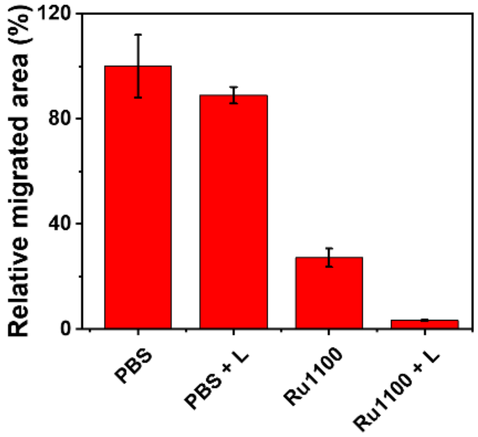

Figure S24. Migration analysis of A549 cells 48 h after treatment under different conditions.

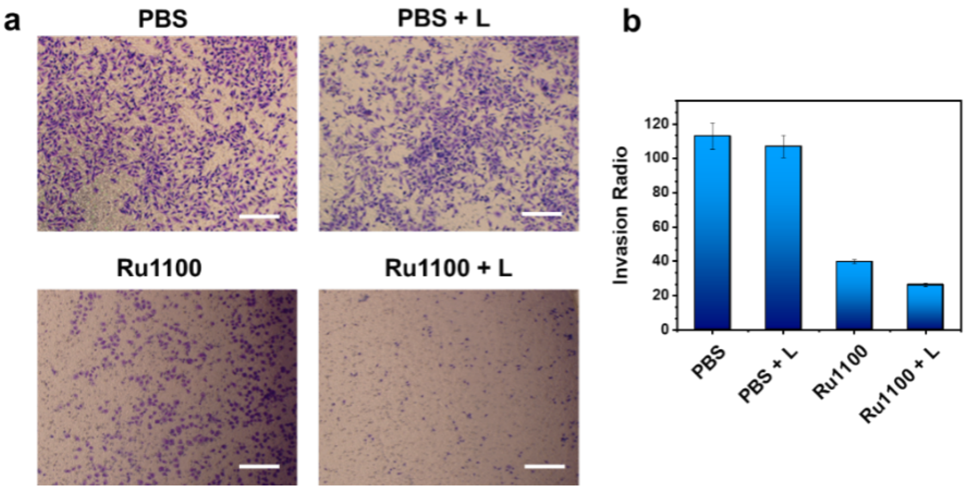

Figure S25. (a) A transwell invasion assay showing the invasion inhibition of A549 by **Ru1100**. Cells were treated with 5 μM **Ru1100** for 24 h. Scale bars = 200 μm. (b) Semi-quantitative graph of (a).

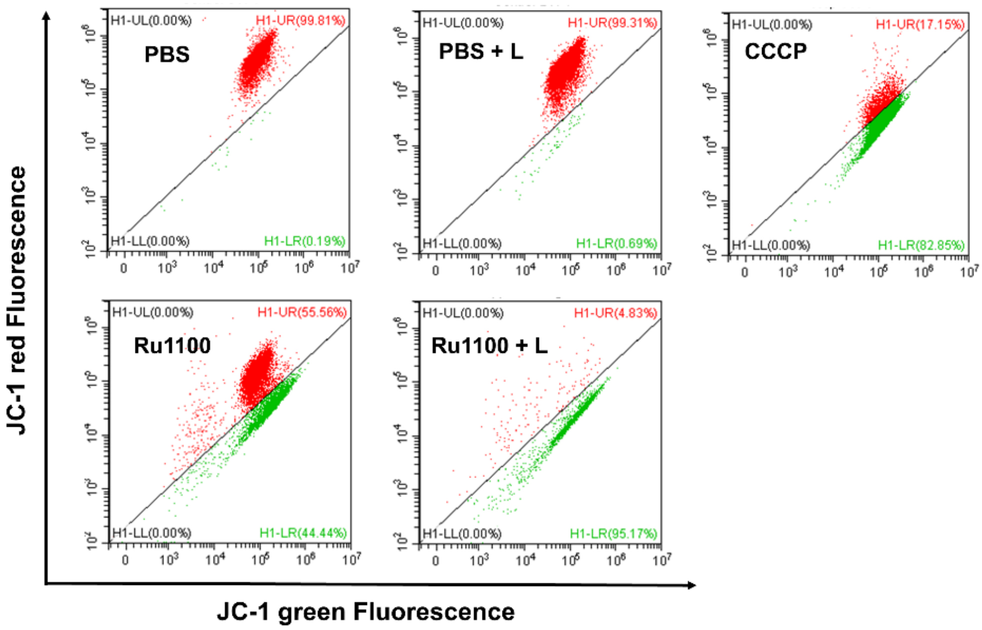

Figure S26. Flow cytometric analysis of A549 cells incubated with **Ru1100** (5 μM) and JC-1 in the absence and presence of light.

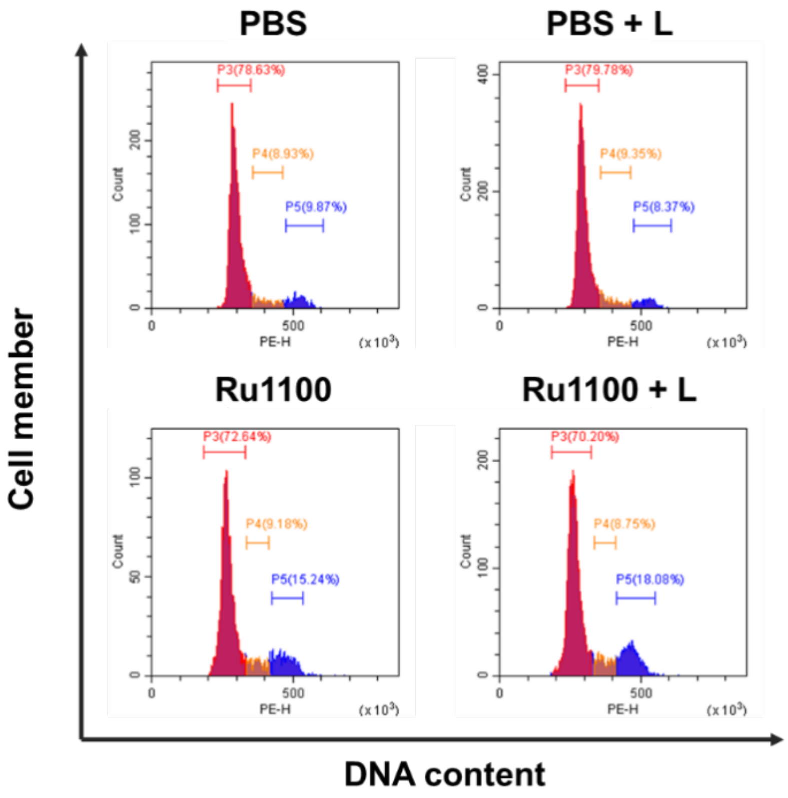

Figure S27. Cell cycle analysis of A549 cells by flow cytometry after incubation with PBS, PBS + L, **Ru1100** and **Ru1100** + L. Here, as elsewhere,. L = laser irradiation at 808 nm.

S5. *In vivo* fluorescence imaging-guided synergistic chemo-phototherapy.

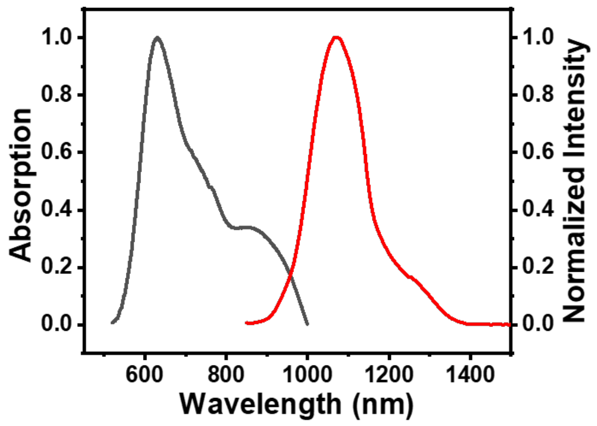

Figure S28. Noremalized UV-Vis-NIR absorption and fluorescence emission of **Ru1100** NPs in H_2O.

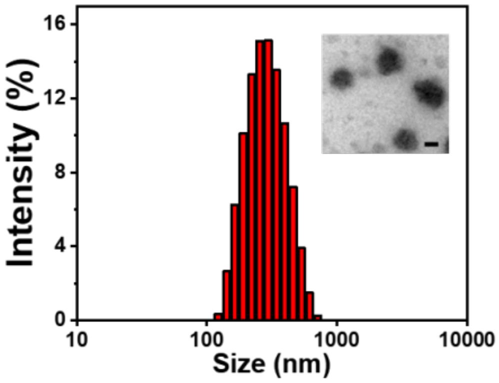

Figure S29. DLS measurements and TEM image of **Ru1100** NPs. Scale bar: 100 μm.

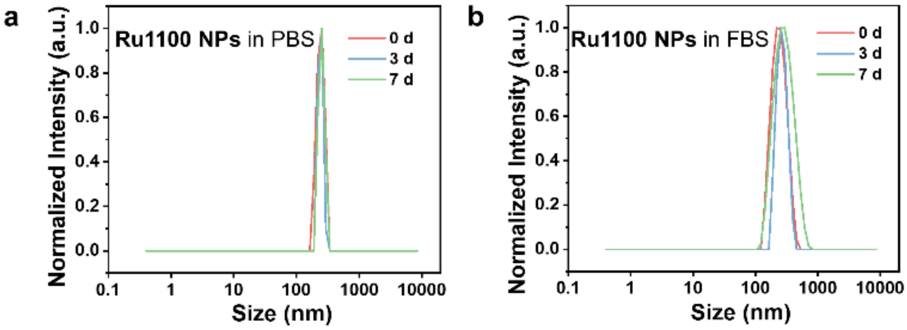

Figure S30. (a) DLS measurements of **Ru1100** NPs in PBS and (b) 10% FBS at various times.

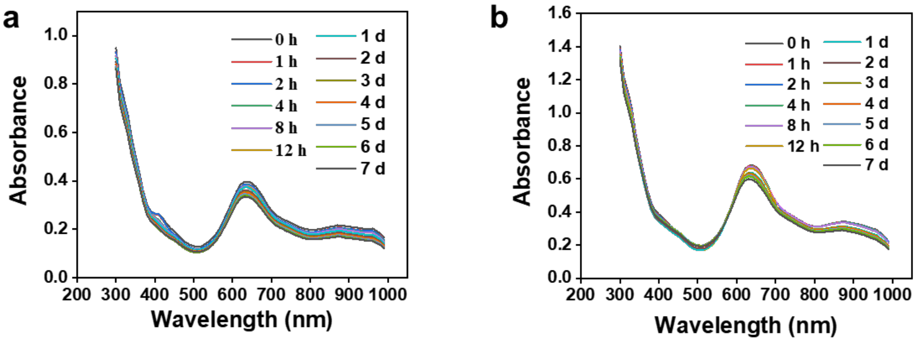

Figure S31. (a) Absorption studies of **Ru1100** NPs in PBS and (b) 10% FBS at various periods.

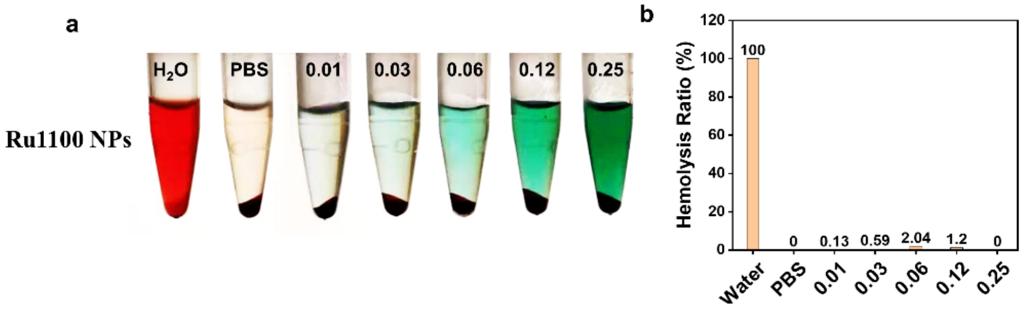

Figure S32. (a) Hemolysis of blood samples treated with different concentrations of **Ru1100 NPs** (0-0.25 μM). (b) Hemolysis ratios based on the study shown in (a).

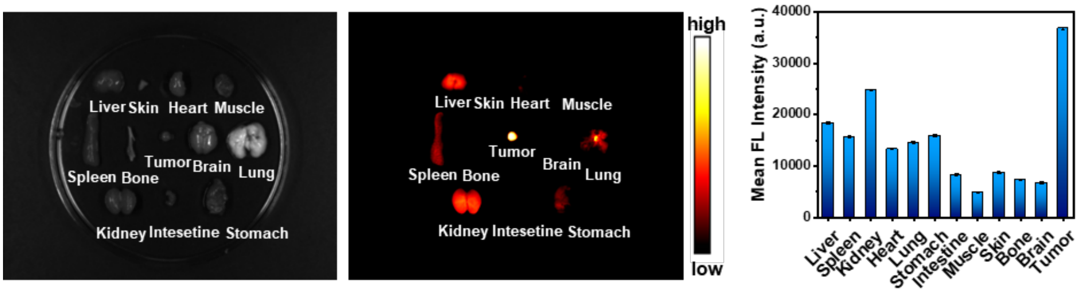

Figure S33. *Ex vivo* biodistribution of A549 tumour-bearing mice as recorded 36 hour post-injection under 808 nm laser illumination (28 mW/cm^2, 1000 LP and 30 ms).

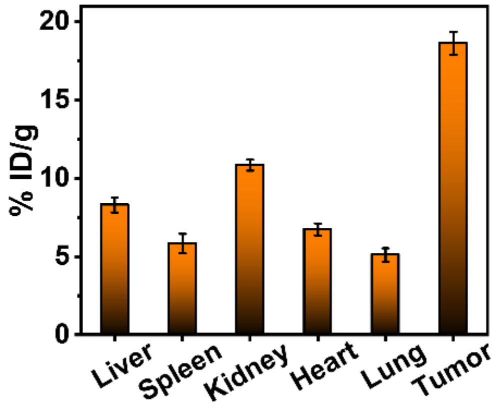

Figure S34. Tissue distributions determined by ICP-MS at 36 h post-injection of **Ru1100** NPs.

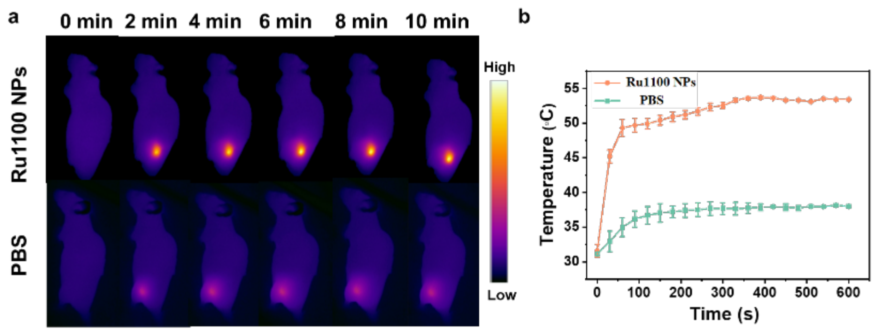

Figure S35. (a) Whole body IR images of mice recorded 24 h after injection with **Ru1100** NPs and PBS and subject to 808 nm laser irradiation (0.8 W/cm^2) and (b) variation in tumour temperature as a function of the laser irradiation time in (a).

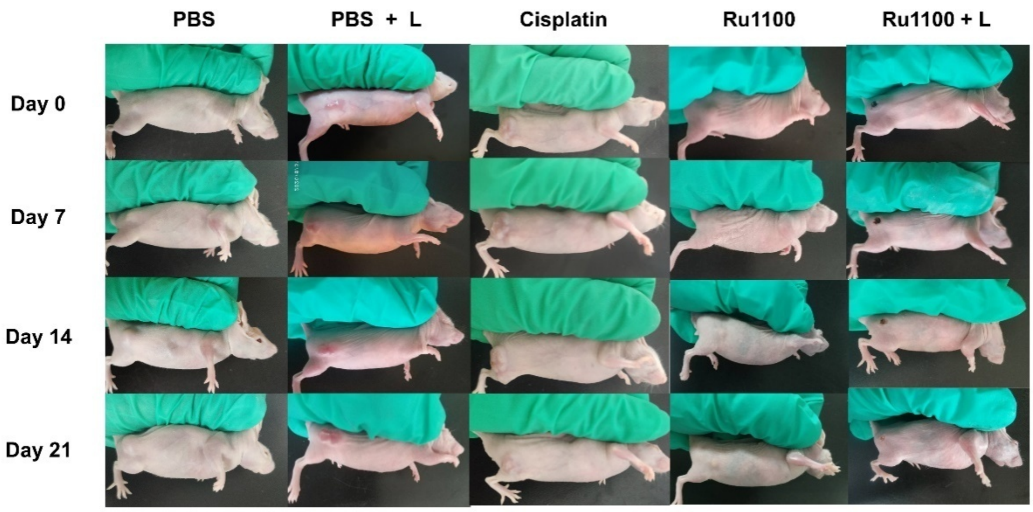

Figure S36. Bright images of mice treated with the indicated formulations at 0, 7, 14 and 21 days.

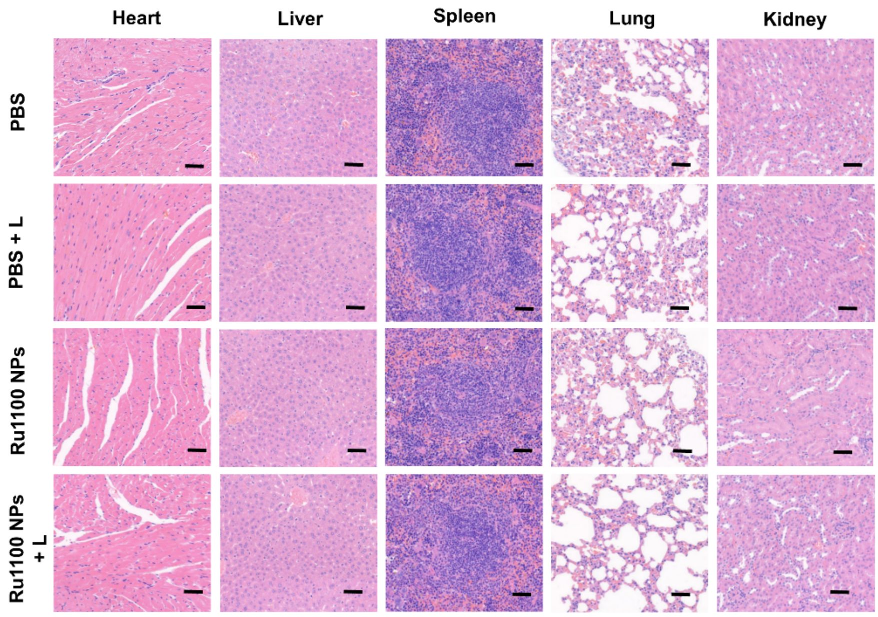

Figure S37. Histological analysis. H&E staining of major organs including the heart, liver, spleen, lungs and kidneys of mice treated with different formulations (PBS, PBS + laser, **Ru1100** NPs and **Ru1100** NPs + laser). Scale bar: 50 μm.

S6. Synthetic procedures and compound characterization.

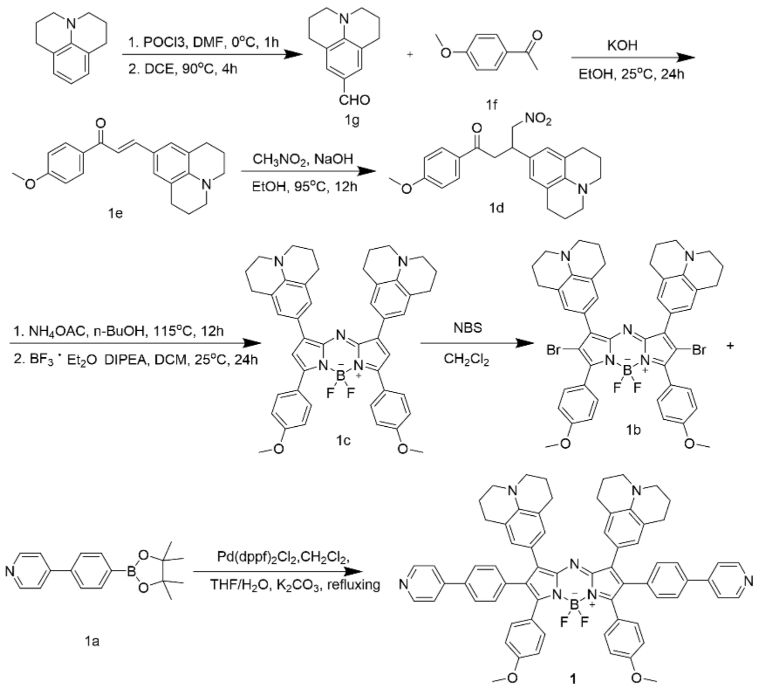

Scheme S1. Synthesis of **Ru1100**.

Synthesis of compound **1g**:

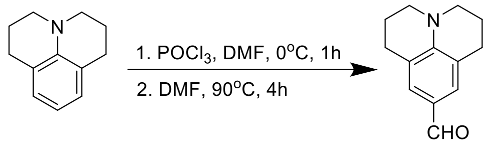

1g

DMF (1.5 mL) was added to freshly distilled $POCl_3$ (1.5 mL) under an atmosphere of N_2 at 0°C and the mixture was stirred at room temperature for 1 h. After the dropwise addition of Julolidine (4.0 g, 23.08 mmol) in DMF (10 mL), the mixture was stirred at room temperature overnight and then poured into a aqueous solution of $NaHCO_3$. After stirring for 2 h at room temperature, the mixture was extracted with ethyl acetate (EA), and the organic fractions were collected and dried over anhydrous Na_2SO_4. The crude product obtained in this way was purified by silica gel chromatography (petroleum ether (PE): EA = 10: 1) to afford a yellow solid **1g** (4.2 g, 90.5% yield). ^{1}H NMR (400 MHz, $CDCl_3$) δ 9.57 (s, 1H), 7.26 (s, 2H), 3.28 – 3.25 (m, 4H), 2.73 (t, *J* = 6.3 Hz, 4H), 1.92 (dd, *J* = 12.0, 6.0 Hz, 4H). ^{13}C NMR (100 MHz, $CDCl_3$) δ 190.13, 147.87, 129.48, 123.94, 120.29, 50.02, 27.64, 21.23.

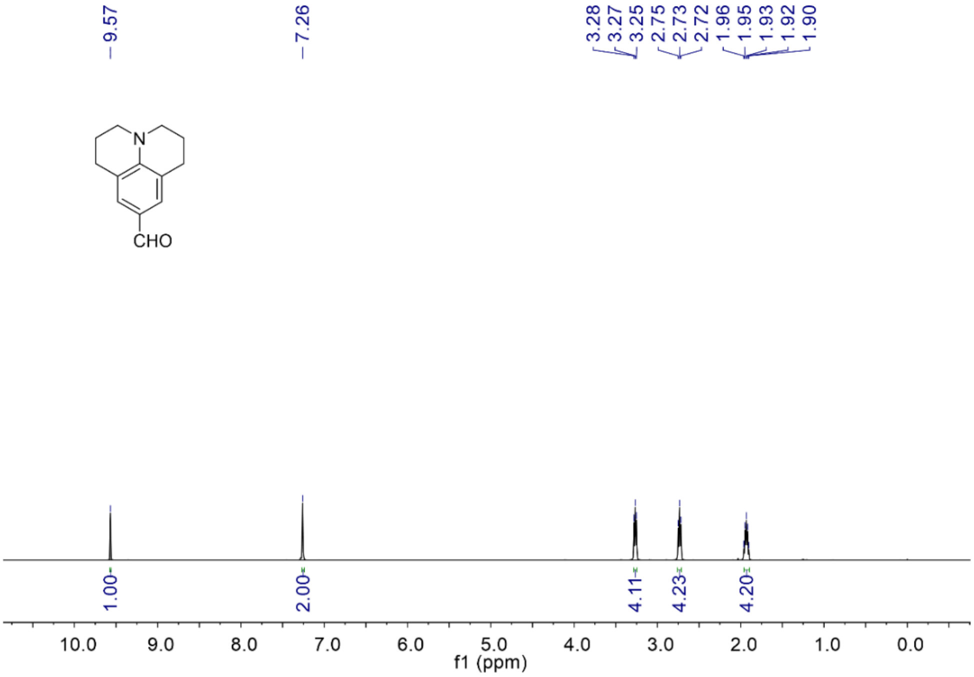

Figure S38. ^{1}H NMR spectrum (400 MHz, $CDCl_3$, 298 K) of **1g**.

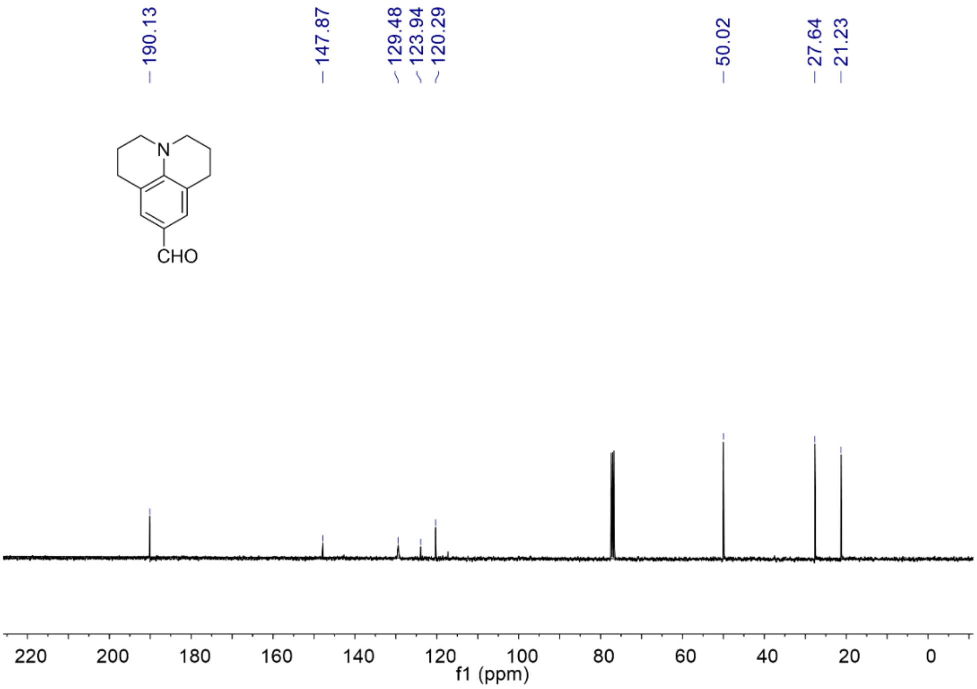

Figure S39. ^{13}C NMR spectrum (100 MHz, $CDCl_3$, 298 K) of **1g**.

Synthesis of compound **1e**:

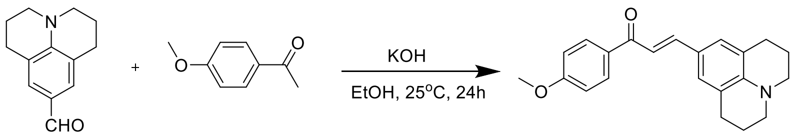

1g 1f 1e

Compound **1g** (4 g, 19.88 mmol) and compound **1f** (2.98 g, 19.88 mmol) were dissolved in EtOH (60 mL). Potassium hydroxide (5.5 g, 99.36 mmol) was then added and the reaction mixture was stirred at room temperature for 24 h. The EtOH and other volatiles were removed under reduced pressure and the resultant aqueous solution was diluted with brine and extracted with EA. The combined organic fractions were dried over anhydrous Na_2SO_4. The organic phase was subject to filteration before being concentrated under reduced pressure to afford a crude residue that was purified *via* flash chromatography on a silica gel column (PE: EA = 15: 1) to afford **1e** as a red solid (5.8 g, 87.6% yield). ^1H NMR (400 MHz, $CDCl_3$) δ 8.02 (d, J = 8.8 Hz, 2H), 7.70 (d, J = 15.3 Hz, 1H), 7.28 (d, J = 16.0 Hz, 1H), 7.11 (s, 2H), 6.96 (d, J = 8.8 Hz, 2H), 3.87 (s, 3H), 3.26 – 3.21 (m, 4H), 2.76 (t, J = 6.3 Hz, 4H), 1.99 – 1.93 (m, 4H). ^{13}C

NMR (100 MHz, $CDCl_3$) δ 188.84, 162.83, 145.52, 145.13, 132.08, 130.48, 128.07, 121.74, 121.00, 115.43, 113.61, 55.46, 49.96, 27.71, 21.59.

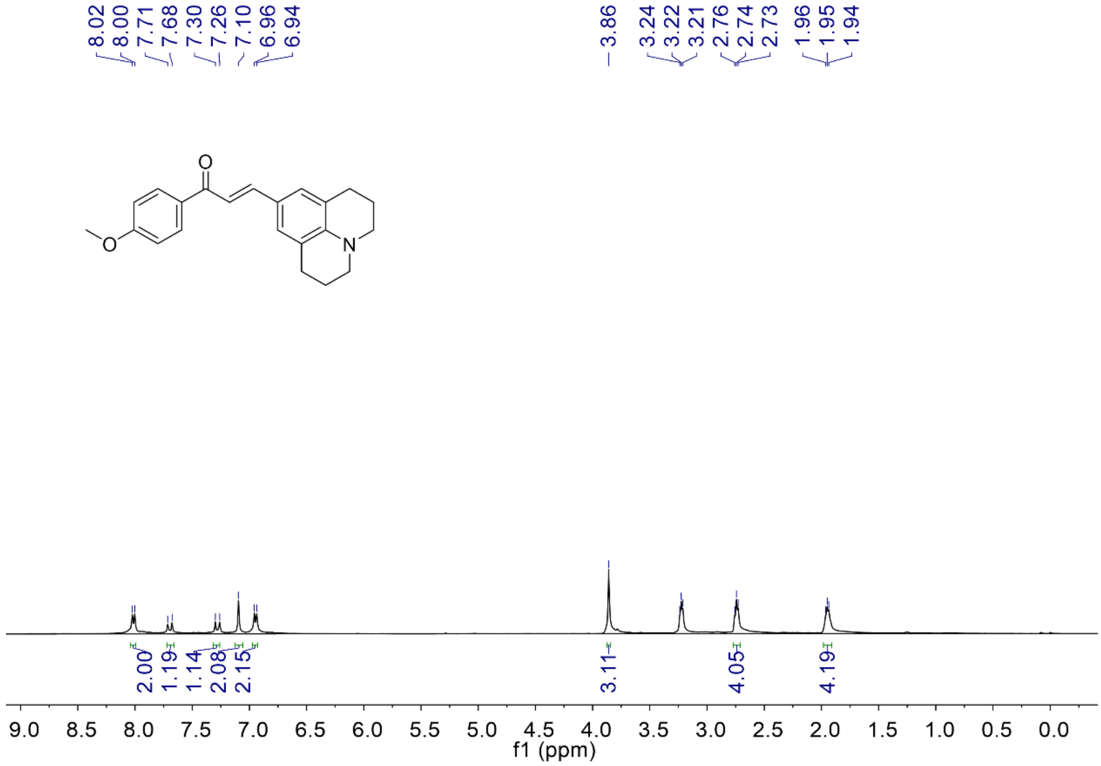

Figure S40. ^1H NMR spectrum (400 MHz, $CDCl_3$, 298 K) of **1e**.

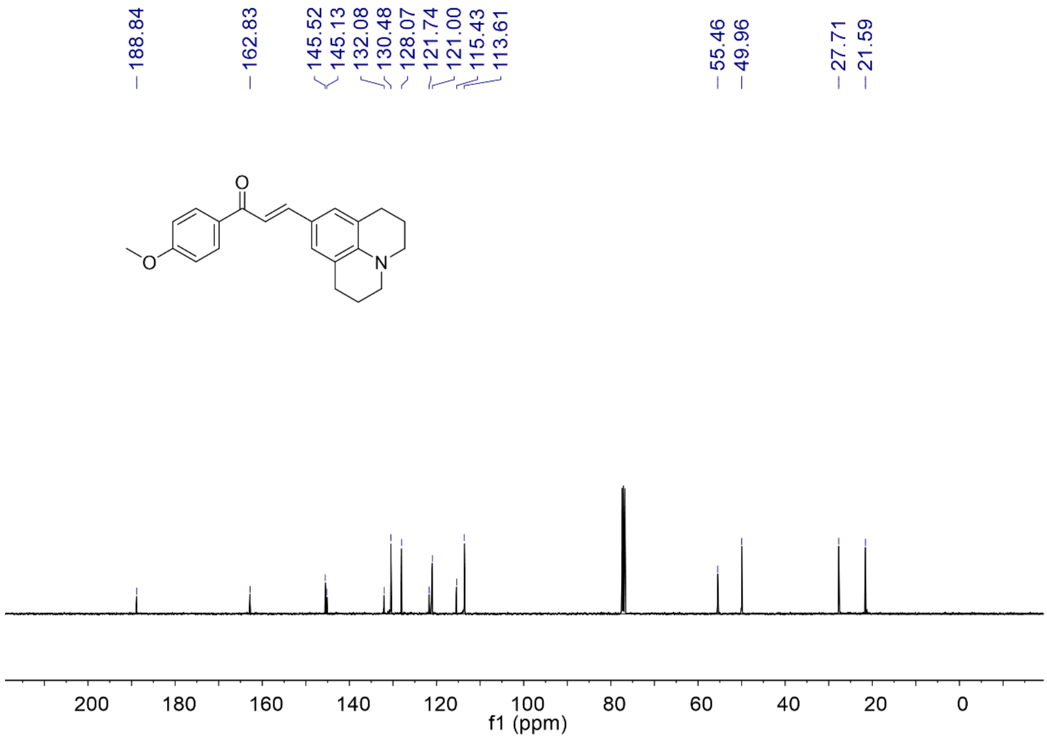

Figure S41. ^{13}C NMR spectrum (100 MHz, $CDCl_3$, 298 K) of **1e**.

Synthesis of compound **1d**:

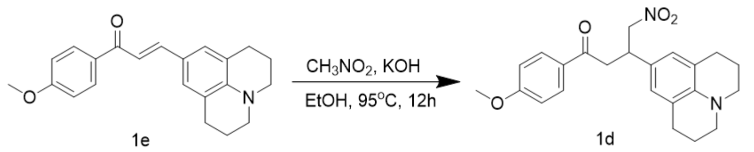

Compound **1e** (1 g, 3.00 mmol) was stirred in EtOH (10 mL) to which solution was added CH_3NO_2 (2.75 g, 44.99 mmol) and potassium hydroxide (168.27 g, 3.00 mmol). The reaction was heated at reflux for 24 h. The EtOH and other volatiles were removed under reduced pressure and the resultant aqueous solution was diluted with brine (20 mL) and extracted with EA. The combined organic fractions were driedover anhydrous Na_2SO_4, filtered and concentrated to afford a crude residue, which was purified via flash chromatography on a silica gel column (PE: EA=10: 1, v/v) to afford compound **1d** as a tan oil (850 mg, 71.85% yield). ^1H NMR (400 MHz, $CDCl_3$) δ 7.91 (d, J = 8.8 Hz, 2H), 6.92 (d, J = 8.8 Hz, 2H), 6.64 (s, 2H), 4.74 (dd, J = 12.2, 6.7 Hz, 1H), 4.58 (dd, J = 12.1, 7.8 Hz, 1H), 4.04 – 3.91 (m, 1H), 3.87 (s, 3H), 3.39 – 3.26 (m, 2H), 3.13 – 3.06 (m, 4H), 2.70 (t, J = 6.4 Hz, 4H), 1.94 (dd, J = 11.7, 6.1 Hz, 4H). ^{13}C NMR (100 MHz, $CDCl_3$) δ 196.08, 163.72, 142.42, 130.41, 129.68, 126.00, 125.83, 121.85, 113.82, 80.07, 55.52, 49.92, 41.61, 38.76, 27.65, 21.95.

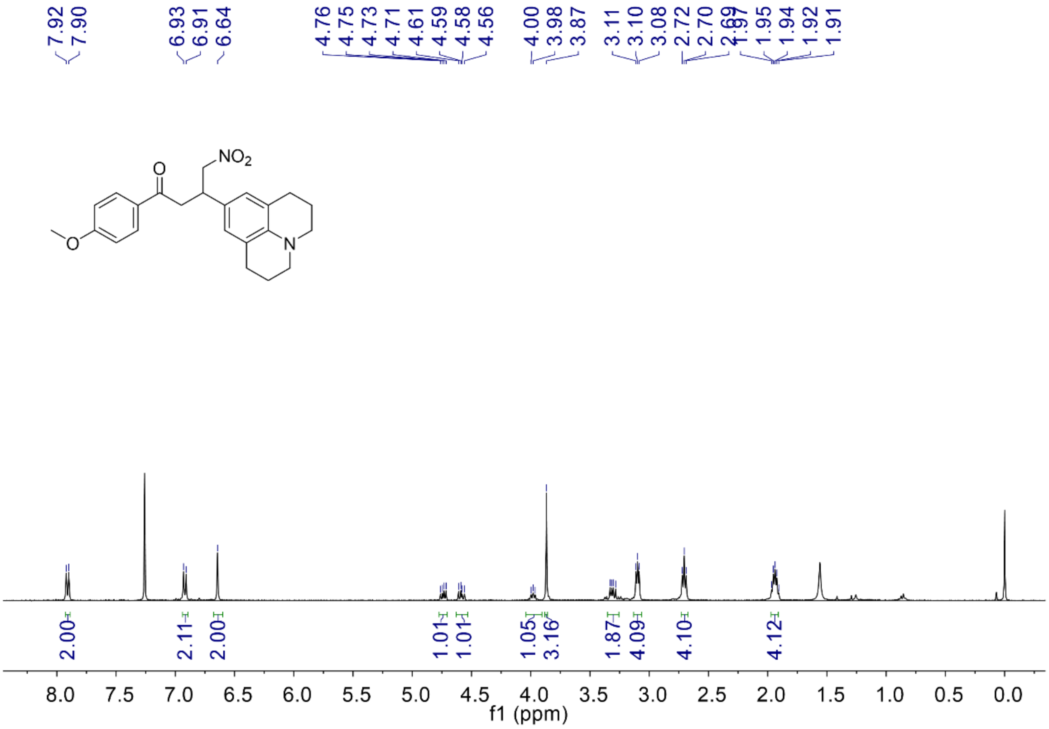

Figure S42. ^{1}H NMR spectrum (400 MHz, $CDCl_3$, 298 K) of **1d**.

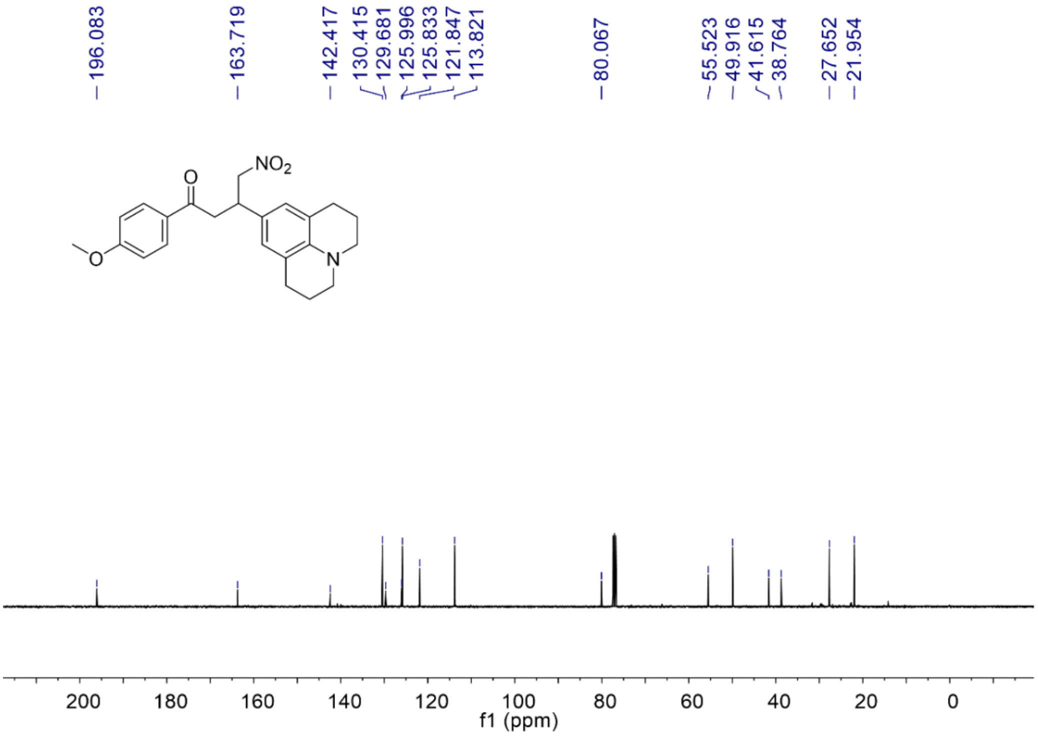

Figure S43. ^{13}C NMR spectrum (100 MHz, $CDCl_3$, 298 K) of **1d**.

Synthesis of compound **1c**:

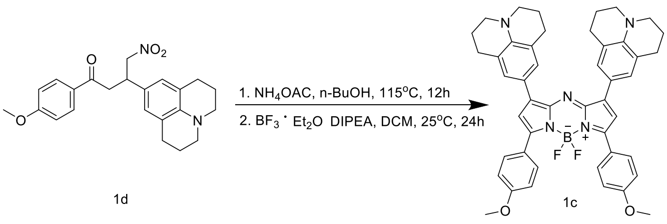

Compound **1d** (850 mg, 2.15 mmol) was dissolved in 1-butanol (20 mL). Ammonium acetate (2.49 g, 32.25 mmol) was added and the reaction was stirred at 115 °C for 12 h. The reaction was cooled to room temperature and the volume was taken to 5 mL under reduced pressure. The reaction mass was then filtered. The isolated solid was washed with ethanol (2× 5 mL) to afford a dark blue film. The film (200 mg, 0.285 mmol) was dissolved in anhydrous DCM (15 mL) and treated with anhydrous N,N-diisopropylethylamine (DIPEA) (421.8 mg, 3.26 mmol, 0.57 mL) and boron trifluoride etherate (608.35 mg, 4.29 mmol, 0.54 mL) and then stirred at room temperature under N_2 for 24 h. The resulting solution was diluted with water (20 mL) and extracted with DCM. The combined organic fractions were dried over anhydrous Na_2SO_4, filtered and concentrated under reduced pressure to afford a crude residue, which was purified via flash chromatography on a silica gel column (PE: DCM=1: 1, v/v) to afford **1c** as a metallic brown solid (190 mg, 88.93% yield). ^{1}H NMR (400 MHz, $CDCl_3$) δ 8.05 (d, J = 8.8 Hz, 4H), 7.64 (s, 4H), 7.01 (d, J = 8.8 Hz, 4H), 6.77 (s, 2H), 3.91 (s, 6H), 3.36 – 3.28 (m, 8H), 2.82 (t, J = 6.0 Hz, 8H), 2.08 – 2.01 (m, 8H). ^{13}C NMR (100 MHz, $CDCl_3$) δ 160.97, 156.00, 145.17, 143.88, 131.08, 128.55, 125.33, 121.17, 120.81, 114.37, 113.85, 55.33, 50.14, 28.03, 21.82.

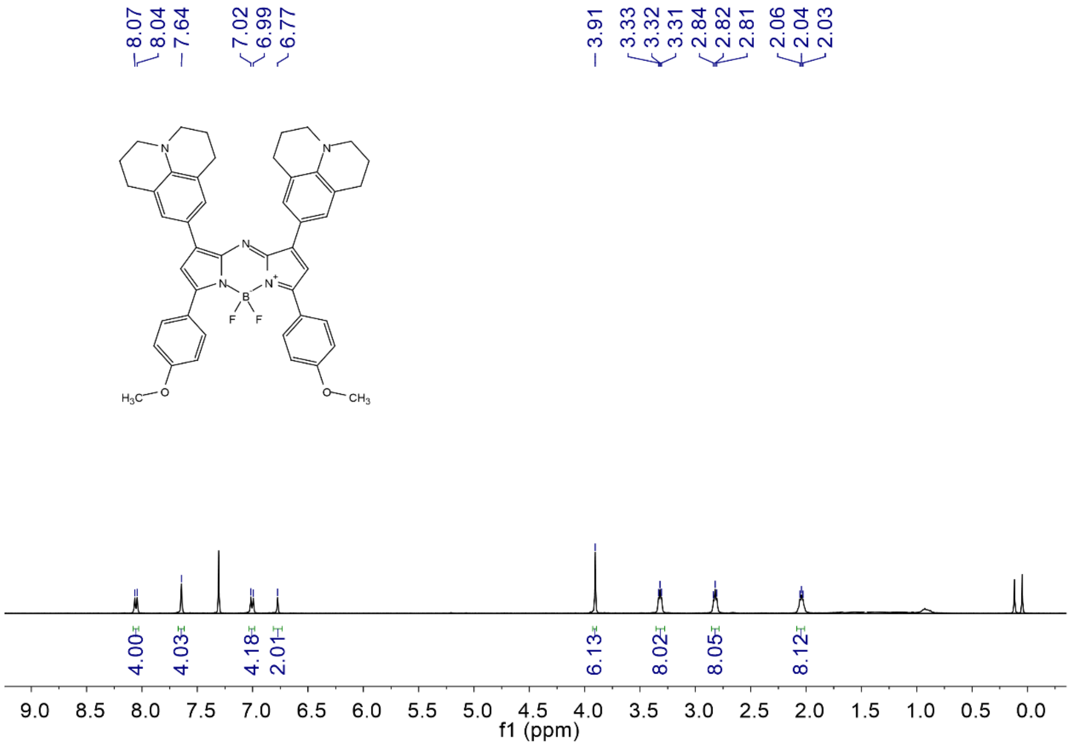

Figure S44. ^{1}H NMR spectrum (400 MHz, $CDCl_3$, 298 K) of **1c**.

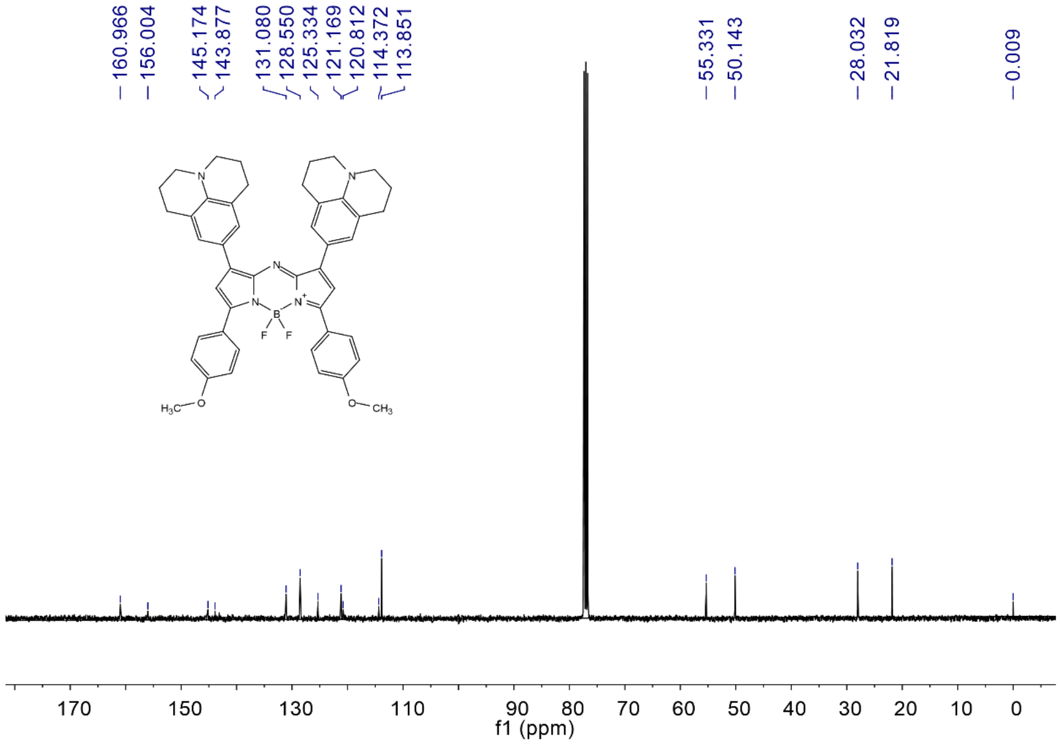

Figure S45. ^{13}C NMR spectrum (100 MHz, $CDCl_3$, 298 K) of **1c**.

Synthesis of compound **1b**:

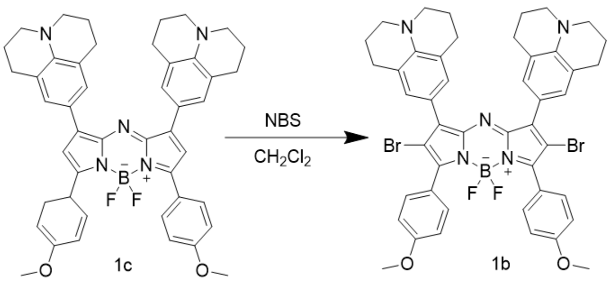

Compound **1c** (56 mg,0.074 mmol) was dissolved in dichloromethane, and then *N*-bromosuccinimide (NBS; 4.25 mg, 0.074 mmol) was added while cooling in an ice bath. After 15 minutes, the ice bath was removed, and the reaction was allowed to proceed at room temperature for 1 h. After diluting with dichloromethane (50 mL), the organic layer was washed with a saturated aqueous NH_4Cl solution (50 mL), water (4 × 50 mL) and saturated aqueous brine (100 mL). It was then dried over anhydrous magnesium sulfate and filtered as well as concentrated to dryness. Purification of the crude product by silica gel chromatography (PE: DCM = 1: 1, v / v) afforded **1b** as a yellow oil (62 mg, 91.6% yield). ^{1}H NMR (400 MHz, $CDCl_3$) δ 7.74 (d, J = 7.8 Hz, 4H), 7.58 (s, 4H), 6.99 (d, J = 7.9 Hz, 4H), 3.88 (s, 6H), 3.30 (s, 8H), 2.83 (s, 8H), 2.03 (s, 8H). ^{13}C NMR (100 MHz, $CDCl_3$) δ 160.89, 144.25, 132.16, 130.18, 122.91, 120.65, 119.09, 113.30, 77.36, 77.05, 76.73, 55.21, 50.11, 27.78, 21.83.

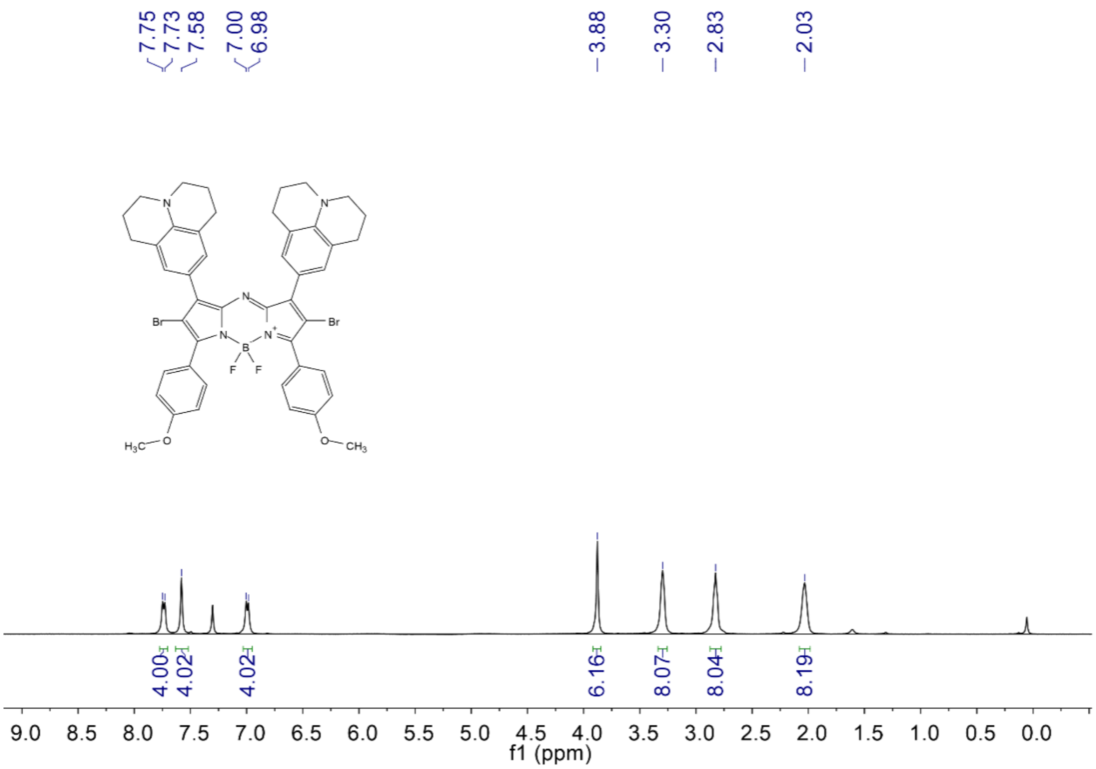

Figure S46. ^{1}H NMR spectrum (400 MHz, $CDCl_3$, 298 K) of **1b**.

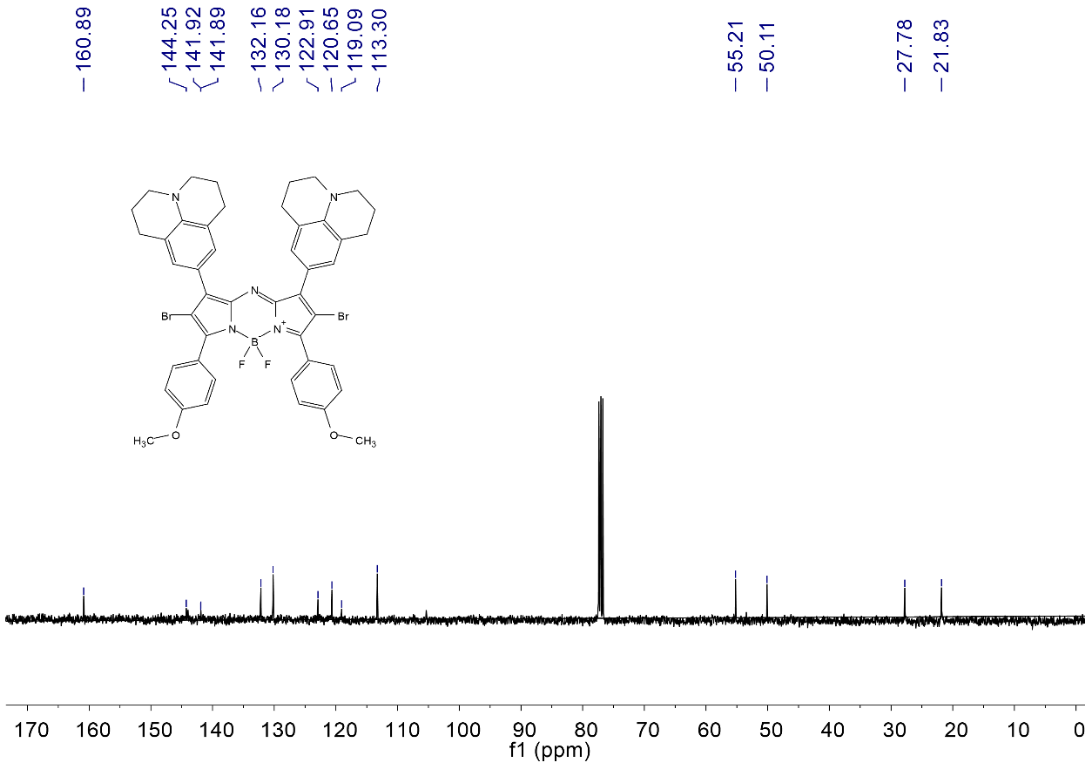

Figure S47. ^{13}C NMR spectrum (100 MHz, $CDCl_3$, 298 K) of **1b**.

2. Synthesis and characterization of **2**

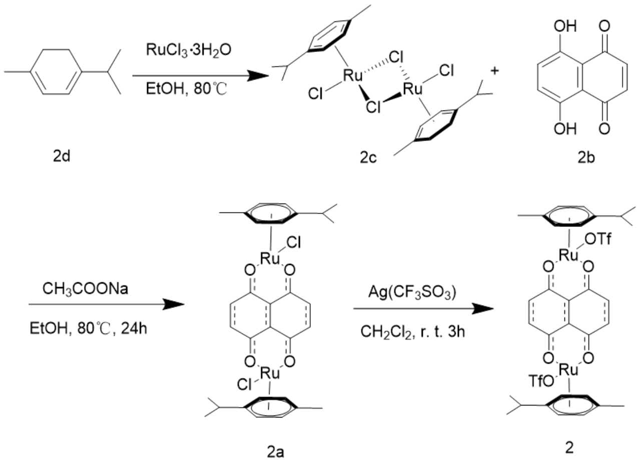

Scheme S2. Synthesis of **2**

Synthesis of compound **2c**:

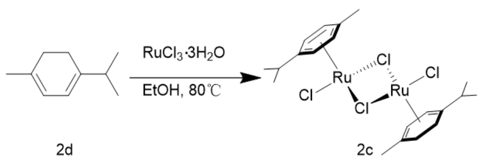

Ruthenium (III) chloride trihydrate (2 g, 7.66 mmol) was dissolved in absolute ethanol, and then compound **2d** (4.57 mL, 3.65 g, 26.8 mmol) was added under an argon atmosphere. The mixture was heated in an oil bath at 80°C for 6 h. After cooling, the reaction mixture was concentrated, subject to suction filteration and washed with ethanol continuously to give product **2c** as a red solid. (1.5 g, 65.2% yield). ^{1}H NMR (400 MHz, $CDCl_3$) δ 5.49 (s, 4H), 5.34 (s, 4H), 2.92 (dt, *J* = 13.9, 6.9 Hz, 2H), 2.16 (s, 6H), 1.28 (d, *J* = 6.9 Hz, 12H). ^{13}C NMR (100MHz, $CDCl_3$) δ 101.19, 96.73, 81.30, 80.54, 30.61, 22.14, 18.91.

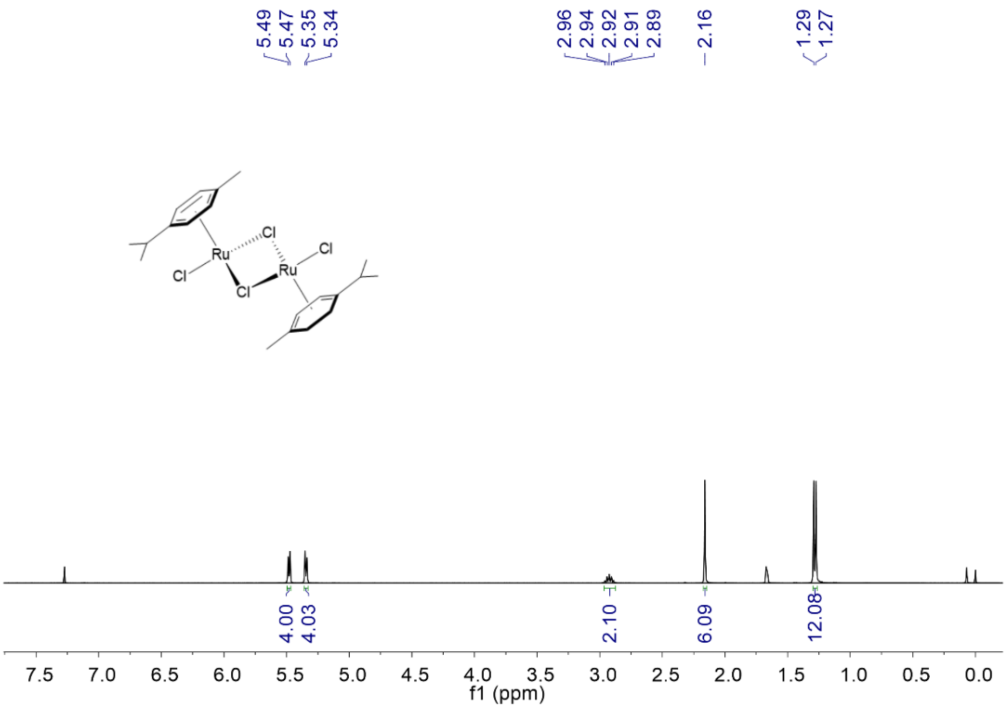

Figure S48. ^{1}H NMR spectrum (400 MHz, $CDCl_3$, 298 K) of **2c**.

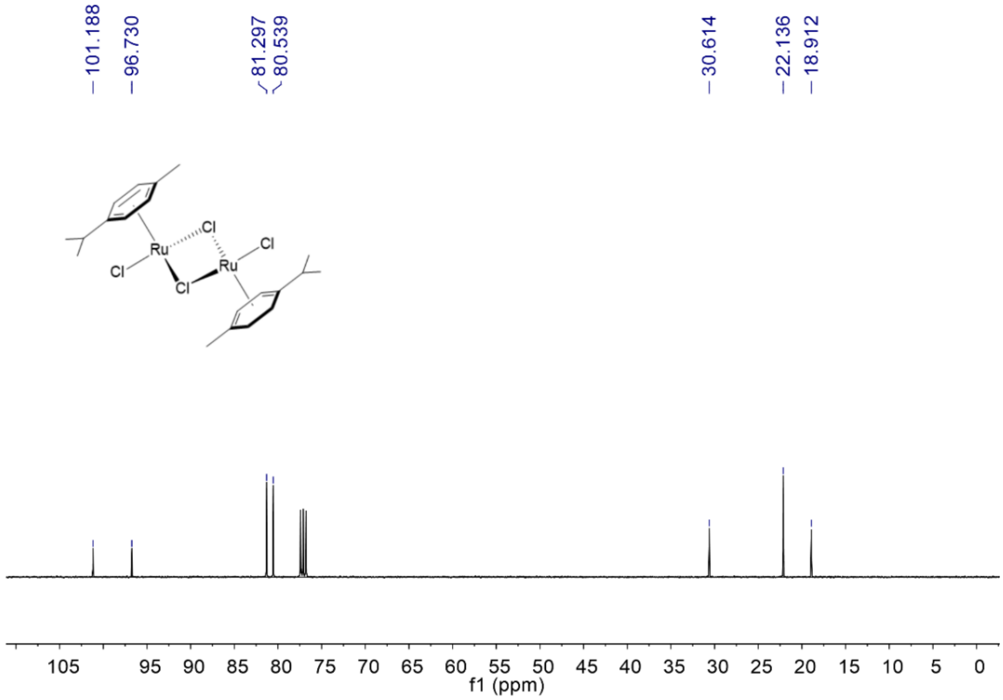

Figure S49. ^{13}C NMR spectrum (100 MHz, $CDCl_3$, 298 K) of **2c**.

Synthesis of compound 2a:

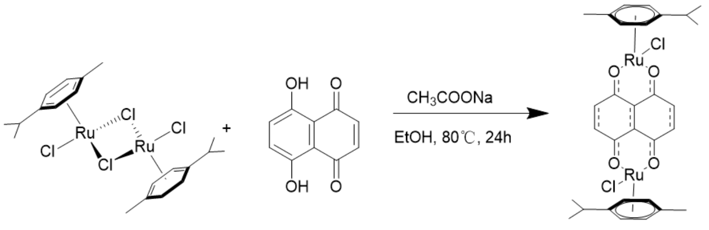

2c 2b 2a

Compound **2c** (0.49 g, 0.80 mmol), sodium acetate (0.14 g,1.71 mmol) and compound **2b** (0.18g, 0.95 mmol) were added to absolute ethanol (20.0 mL) under an argon atmosphere. The mixture was heated in an oil bath at 80°C for 24 h. After allowing to cool, the reaction mixture was concentrated under reduced pressure, subject to suction filteration and washed with ethanol, water, acetone, and ether continuously to give product **2a** as a black solid (0.57 g, 97.5% yield). ^{1}H NMR (400 MHz, $CDCl_3$) δ 6.99 (s, 4H), 5.53 (d, J = 5.7 Hz, 4H), 5.27 (d, J = 5.6 Hz, 4H), 2.95 – 2.87 (m, 2H), 2.26 (s, 6H), 1.35 (d, J = 6.9 Hz, 12H). ^{13}C NMR (100 MHz, $CDCl_3$) δ

171.01, 137.07, 111.98, 100.39, 98.00, 82.89, 79.68, 30.79, 22.39, 17.93.

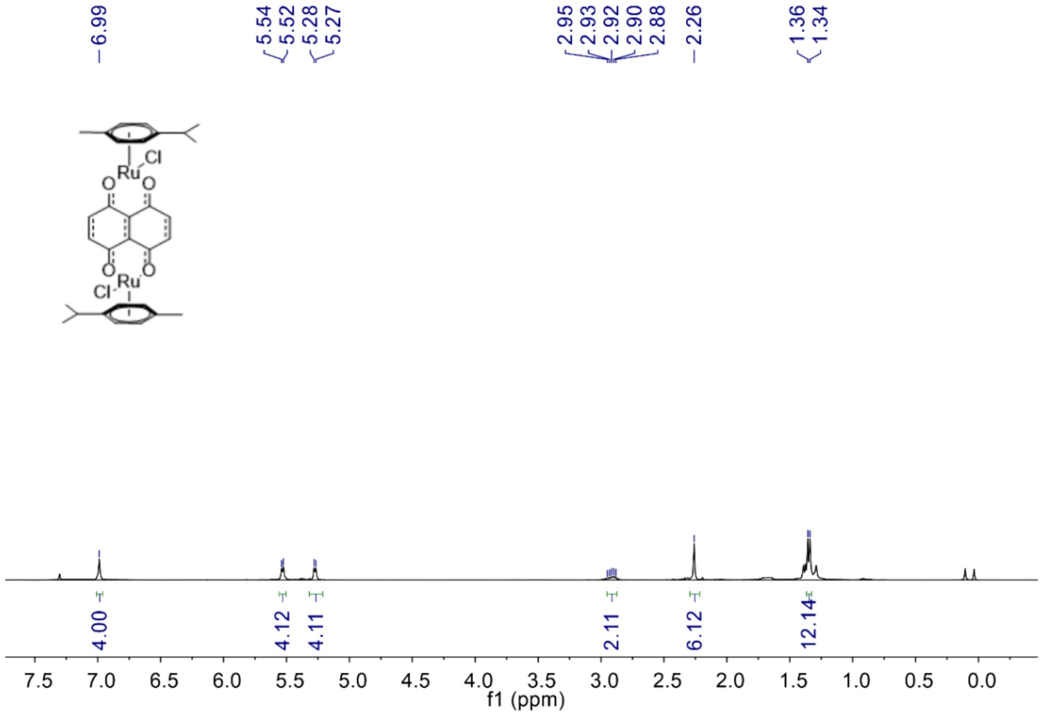

Figure S50. ^{1}H NMR spectrum (400 MHz, $CDCl_3$, 298 K) of **2a**.

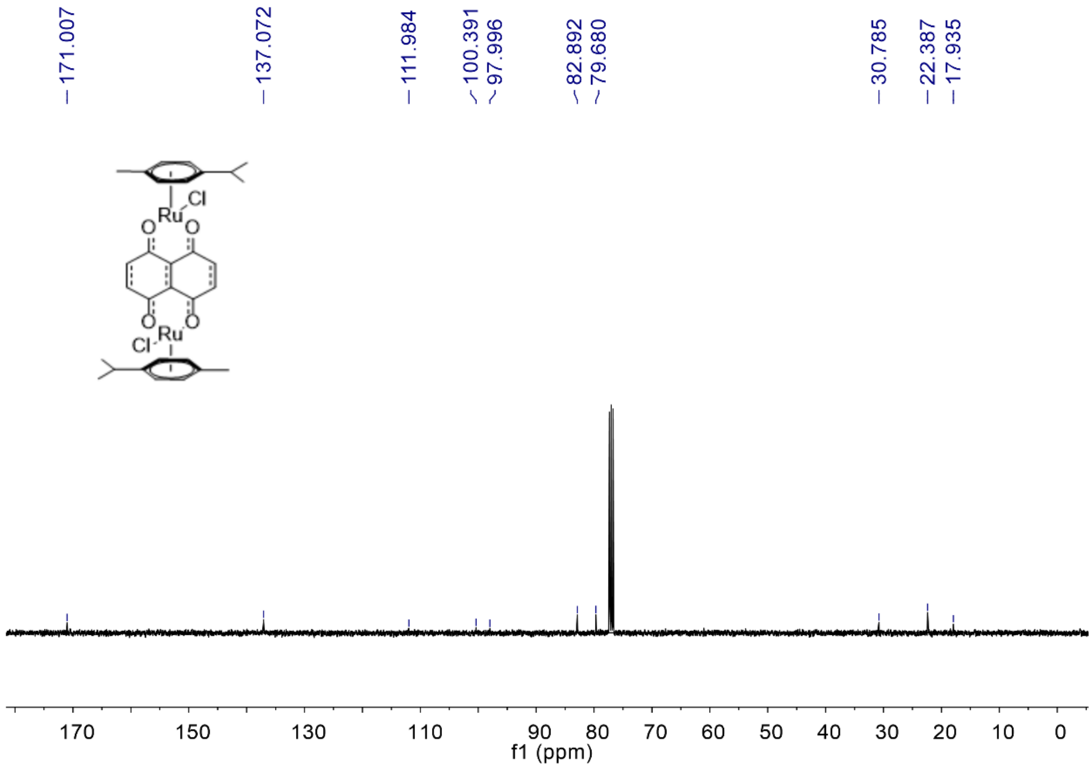

Figure S51. ^{13}C NMR spectrum (100 MHz, $CDCl_3$, 298 K) of **2a**.

Synthesis of compound 2:

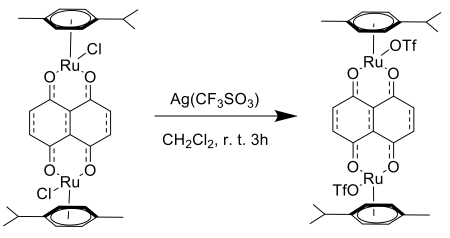

2a 2

Compound **2a** (0.57 g, 0.78 mmol) and silver trifluoromethanesulfonate (0.41 g, 1.60 mmol) were dissolved in DCM and stirred at room temperature for 3 h. The black-green reaction solution was subject to suction filteration and washed with methanol to give product **2** (0.480 g, 84.4% yield). ^{1}H NMR (400 MHz, CD_3OD) δ 7.24 (s, 4H), 5.78 (d, J = 6.2 Hz, 4H), 5.53 (d, J = 6.2 Hz, 4H), 2.80 (t, J = 6.9 Hz, 2H), 2.17 (s, 6H), 1.29 (d, J = 6.9 Hz, 12H). ^{13}C NMR (100 MHz, CD_3OD) δ 171.41, 137.24, 121.96, 118.79, 110.31, 100.86, 98.40, 81.38, 78.85, 30.77, 21.00, 16.23.

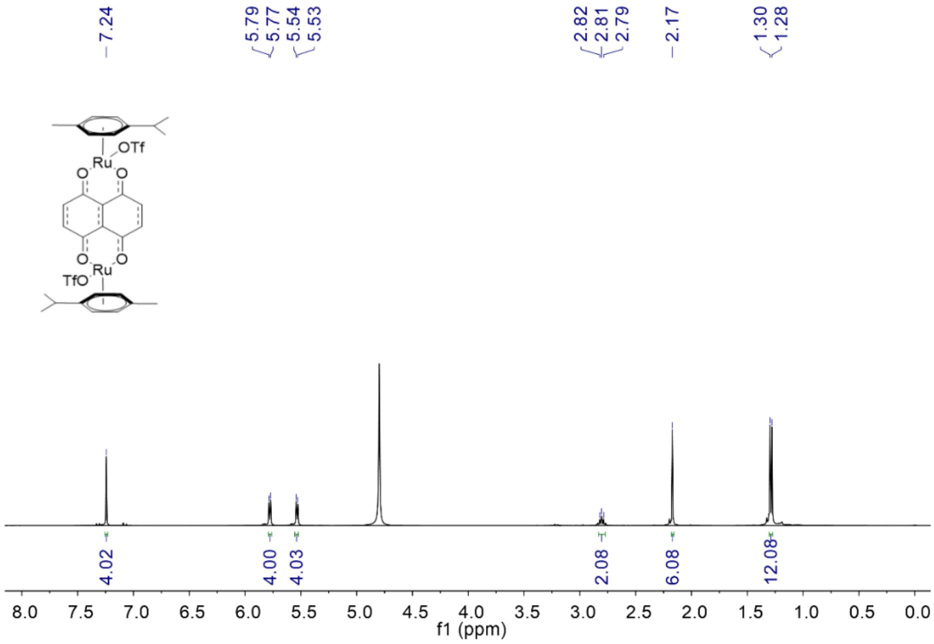

Figure S52. ^{1}H NMR spectrum (400 MHz, CD_3OD, 298 K) of **2**.

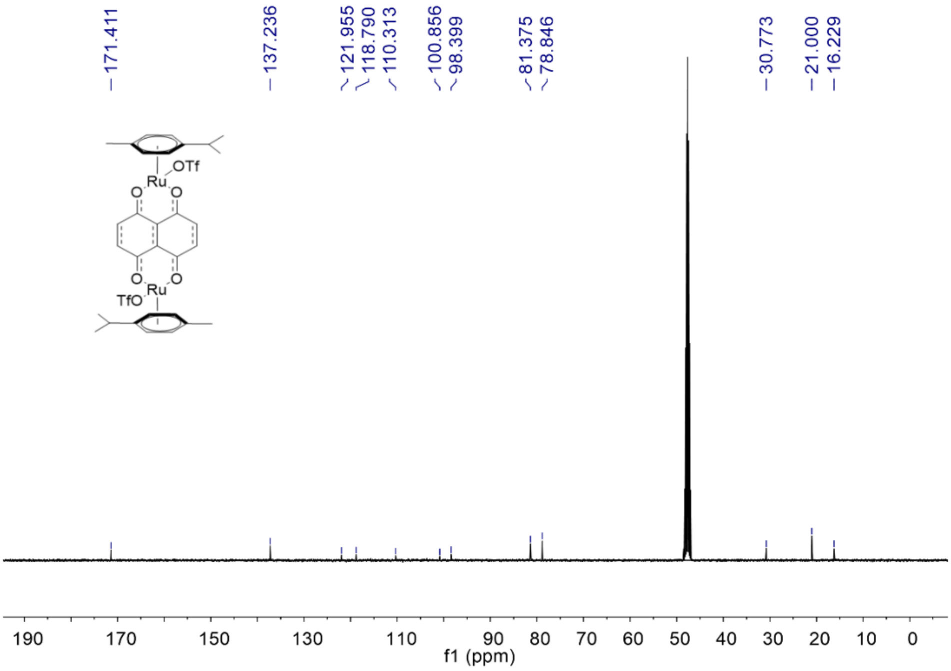

Figure S53. ^{13}C NMR spectrum (100 MHz, CH_3OD, 298 K) of **2**.

S7. Supplementary references

S1. Bai, L.; Sun, P.; Liu, Y.; Zhang, H.; Hu, W.; Zhang, W.; Liu, Z.; Fan, Q.; Li, L.; Huang, W. Novel aza-BODIPY based small molecular NIR-II fluorophores for in vivo imaging. *Chem. Commun*. **2019**, *55*, 10920-10923.

S2. Gomes, A.; Fernandes, E.; Lima, J. L. Fluorescence probes used for detection of reactive oxygen species.*Biochem.Biophys*. *Methods* **2005**, *65*, 45-80.

S3. Baka, E.; Comer, J. E. A.;Takács-Novák, K. Study of equilibrium solubility measurement by saturation shakeflask method using hydrochlorothiazide as model compound. *J. Pharm. Biomed. Anal.* **2008**, *46*, 335-341.

S4. Li. C.;Yu, M.; Sun, Y.; Wu, Y.; Huang, C.; Li, F. A NonemissiveIridium(III) Complex That

Specifically Lights-Up the Nuclei of Living Cells. *J. Am. Chem. Soc.* **2011**, *133*, 11231-11239.

S5. Su, S. J.; Tanaka, D.; Li, Y.; Sasabe, H.; Takeda, T.; Kido, J. Novel four-pyridylbenzene-armed biphenyls as electron-transport materials for phosphorescent OLED. *Org. lett.* **2008**, *10*, 941-944.
